# Targeted labeling and depletion of alveolar macrophages using VeDTR mouse technology

**DOI:** 10.1016/j.isci.2025.111975

**Published:** 2025-02-09

**Authors:** Yuki Nakayama, Miwa Sasai, Ayumi Kuratani, Masaaki Okamoto, Daisuke Okuzaki, Kentaro Yamamoto, Chikako Ono, Masaya Yamaguchi, Shigetada Kawabata, Noriko Shinjyo, Yasutaka Okabe, Yoshiharu Matsuura, Manabu Ato, Masahiro Yamamoto

**Affiliations:** 1 Department of Immunoparasitology, Research Institute for Microbial Diseases, Osaka University, Suita, Osaka 565-0871, Japan; 2 Laboratory of Immunoparasitology, WPI Immunology Frontier Research Center, Osaka University, Suita, Osaka 565-0871, Japan; 3 Center for Infectious Disease Education and Research, Osaka University, Suita, Osaka 565-0871, Japan; 4 Center for Advanced Modalities and Drug Delivery Systems, Osaka University, Suita, Osaka 565-0871, Japan; 5 Laboratory of Human Immunology (Single Cell Genomics), WPI Immunology Frontier Research Center, Osaka University, Suita, Osaka 565-0871, Japan; 6 Bioinformatics Center, Research Institute for Microbial Diseases, Osaka University, Suita, Osaka 565-0871, Japan; 7 Department of Mycobacteriology, Leprosy Research Center, National Institute of Infectious Diseases, Tokyo, Japan; 8 Laboratory of Virus Control, Research Institute for Microbial Diseases, Osaka University, Suita, Osaka 565-0871, Japan; 9 Microbial Research Center for Health and Medicine, National Institutes of Biomedical Innovation, Health and Nutrition, Ibaraki, Osaka 567-0085, Japan; 10 Bioinformatics Research Unit, Osaka University Graduate School of Dentistry, Suita, Osaka 565-0871, Japan; 11 Department of Microbiology, Osaka University Graduate School of Dentistry, Suita, Osaka 565-0871, Japan; 12 Laboratory of Immune Homeostasis, WPI Immunology Frontier Research Center, Osaka University, Suita, Osaka 565-0871, Japan; 13 School of Tropical Medicine and Global Health, Nagasaki University, Sakamoto, Nagasaki 852-8523, Japan

**Keywords:** Biotechnology, Immunology, Microbiology

## Abstract

Alveolar macrophages (AMs) are essential for maintaining lung homeostasis. However, their roles in respiratory infections have been controversial because the methods of depleting them have often suffered from poor cell selectivity. To resolve this problem, we here used VeDTR technology to generate a transgenic mouse line in which AMs can be specifically depleted using diphtheria toxin. When various respiratory infections were examined using this system, we found that AMs prevented the proliferation of *Mycobacterium abscessus*. This result differed from previous findings using clodronate liposomes to deplete macrophages. We also revealed that the disappearance of AMs contributes to the reduction of bacterial load in the lungs and that AMs are indispensable for GM-CSF-mediated defense against *M. abscessus* infection. Taken together, the development of an AM-specific depletion system has provided an opportunity to study the roles of AMs in various respiratory infections from a different perspective.

## Highlights

• We generated mice that can specifically label and deplete alveolar macrophages
• Alveolar macrophages play a role in inhibiting *Mycobacterium abscessus in vivo* growth
• IFN-γ-dependent alveolar macrophages disappearance prevents *M. abscessus* infection
• GM-CSF inhalation acts on alveolar macrophages to reduce *M. abscessus* proliferation

## Graphical abstract

**Figure:**
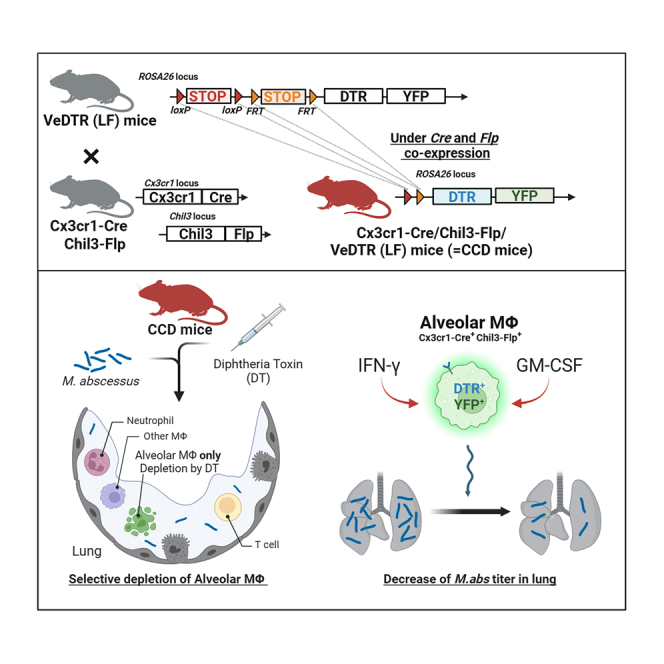

## Introduction

Alveolar macrophages (AMs) are a type of tissue-resident macrophage in the lungs.^1^^,^^2^^,^^3^^,^^4^^,^^5^^,^^6^ Yolk sac–derived AM progenitors mature and self-renew through the influence of granulocyte–macrophage colony-stimulating factor (GM-CSF).^7^^,^^8^ AMs have two main roles: First, they clear commensal bacteria and foreign bodies from the airways; and second, they remove the excess alveolar surfactant produced by the type II alveolar epithelium to prevent alveolar collapse. However, in the lungs of humans with autoantibodies against GM-CSF, dysfunctional AMs fail to remove this surfactant, leading to pulmonary alveolar proteinosis.^9^^,^^10^ This indicates that AMs are essential for respiratory homeostasis. AMs also disappear from the lungs during acute severe inflammation.^4^^,^^11^ Although their disappearance has been shown to involve cell death,^12^ their biological significance in infection and immunity remains unclear.

Several methods for depleting AMs to analyze their function have been reported that can be broadly classified into chemical methods using clodronate liposomes and genetic methods using gene-modified mice. The use of clodronate liposomes is a classic method that can induce apoptosis when these liposomes are taken up by macrophages.^13^ It is not selective for AMs, but can target them upon intranasal administration.^14^ Although clodronate liposomes were originally thought to be highly selective for macrophages, a recent report revealed that neutrophils phagocytosing these liposomes are also stunned and become dysfunctional.^15^ This suggests the need to revisit previous studies using clodronate liposomes to analyze macrophages, including AMs. In terms of the genetic methods for analyzing AMs, these can be further subdivided into those using gene knockout mice and diphtheria toxin receptor (DTR)-expressing mice. Mice lacking GM-CSF or its receptor (Csf2r) have been shown to lack AMs.^16^^,^^17^ These knockout mice exhibit not only the constitutive depletion of AMs but also the depletion of some dendritic cells,^18^ and develop pulmonary alveolar proteinosis after ages 4–6 weeks.^16^^,^^17^ Considering that the accumulated alveolar surfactant itself promotes the phagocytosis of microorganisms,^19^^,^^20^ it remains unclear whether such environmental changes in the lung directly or indirectly affect the bacterial titers there. In terms of the other subcategory, namely, DTR-expressing mice, for analyzing AMs, these include CD11c-DTR mice and CD169Cre-DTR mice. In these mice, DTR expression is controlled by the CD11c promoter directly or by crossing CD169-Cre mice with Rosa26-loxP-DTR mice.^21^^,^^22^^,^^23^^,^^24^ Although these DTR mice exhibit the timely depletion of AMs upon diphtheria toxin (DT) treatment, other myeloid cells are also eliminated. Taken together, these findings suggest a range of difficulties associated with these methods for AM depletion.

Respiratory infections can be caused by many pathogens, including *Streptococcus pneumoniae*, SARS-CoV-2, and various mycobacterial species. Among these, mycobacteria are acid-fast bacteria that initially infect AMs upon entry through the respiratory tract. They then replicate within AMs and disseminate to interstitial macrophages and neutrophils.^25^^,^^26^ The roles of AMs in mycobacterial infection have been studied by chemical or genetic deletion of AMs in *Mycobacterium tuberculosis* (*Mtb*) infection models. When AMs were chemically depleted by clodronate liposomes, *Mtb* titers were reduced,^14^ suggesting that AMs promote the spread of mycobacterial infection. By contrast, when AMs were genetically ablated using GM-CSF-deficient mice or CD11c-DTR mice, *Mtb* titers were increased,^27^^,^^28^ suggesting that AMs inhibit mycobacterial infection. However, because these mice also have dysfunctional dendritic cells and the surfactant proteins accumulating in GM-CSF-deficient mice themselves promote *Mtb* growth,^29^^,^^30^ it is currently controversial whether AMs promote or inhibit mycobacterial infection.

Among *Mycobacterium* spp., *Mycobacterium abscessus* (*M. abs*) is a nontuberculous mycobacterium (NTM) that grows more rapidly and is more difficult to treat than *Mtb* and other NTMs because of the possession of a drug resistance gene, *erm*(41), which confers resistance to macrolide antibiotics.^31^ According to the 2020 ATS/ERS/ESCMID/IDSA guidelines established by four major infectious disease societies,^32^ approximately half of *M. abs* cases do not respond after more than 12 months on guidelines-based therapy, and thus a novel therapy for such infection is urgently needed. As an alternative method to treat refractory NTM infection, GM-CSF inhalation therapy has attracted substantial attention. Indeed, previous clinical case studies demonstrated that inhaled GM-CSF was effective in treating refractory NTM.^33^^,^^34^^,^^35^ Given that GM-CSF plays a role in the *in vivo* development of AMs,^7^ the effectiveness of GM-CSF inhalation therapy against NTM might involve AMs. However, little is currently known about the causal relationship between GM-CSF-dependent anti-NTM therapy and AMs.

In this study, we have generated a mouse line for targeted AM depletion using our previously developed VeDTR mouse system, in which a cell population of interest can be specifically depleted by DT using both *Cre*-*loxP* and *Flp*-*FRT* recombination systems.^36^ We further clarify the role of AMs and the significance of their disappearance during respiratory infections. Moreover, we demonstrate an important role of AMs in inhaled GM-CSF’s effects against NTM.

## Results

### AM-specific YFP expression in Cx3Cr1-Cre/Chil3-Flp/VeDTR (LF) mice

**Figure 1:**
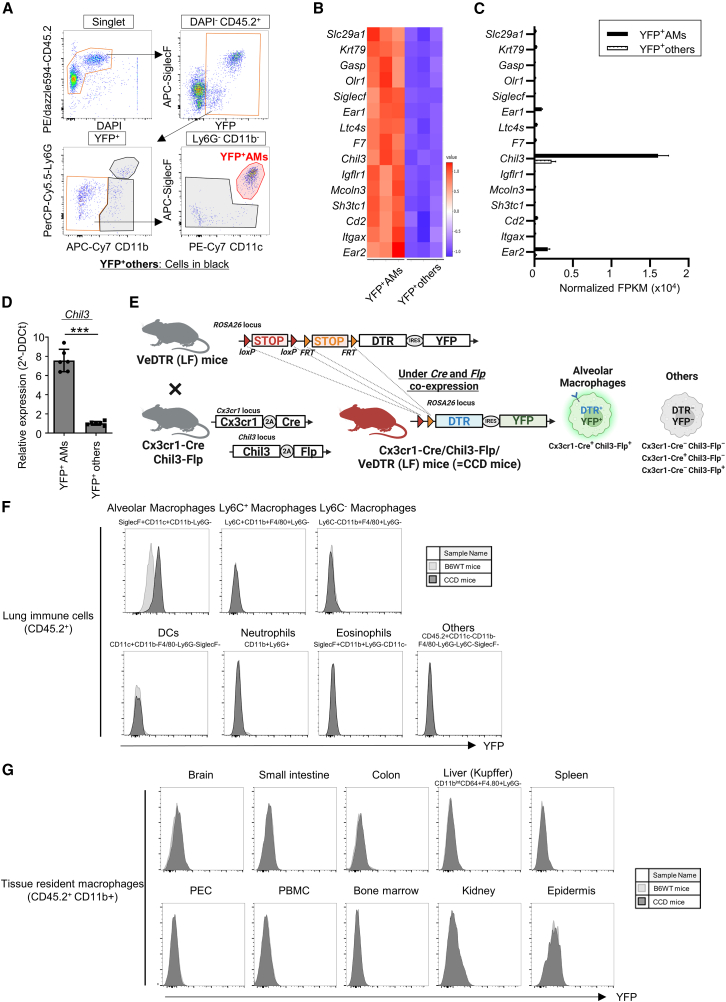
Generation of Cx3cr1-Cre/Chil3-Flp/VeDTR (LF) mice (A) FACS gating strategies. YFP^+^ AMs (red) and YFP^+^ others (black) used for RNA sequencing correspond to YFP^+^ SiglecF^+^ CD11c^+^ CD11b^−^ Ly6G^−^ CD45^+^ cells and the YFP^+^ cell population without AMs, respectively. (B and C) Gene list from RNA sequencing showing higher expression in YFP^+^ AMs (red in A) compared to YFP^+^ others (black in A) in the lungs of Cx3cr1-Cre/VeDTR (ΔFRT) (*n* = 3). The heatmaps are ordered from top to bottom with the most significant differences (B). The normalized FPKMs show the expression levels of each gene (C). This gene list includes genes that are highly expressed in the lung and have low expression levels in other organs, as identified using the BioGPS database of genetic expression profiles. (D) RT-qPCR analysis of *Chil3* gene expression with *β-actin* as the endogenous control in YFP^+^ AMs (red in A) and YFP^+^ others (black in A) in the lungs (*n* = 3 each). Expression level was calculated using the comparative cycle threshold (2^−ΔΔCt^) value method. Data are representative of two independent experiments. Statistical significance assessment: unpaired two-tailed Student’s *t* test. Error bars represent SD.∗∗∗*p* < 0.001. (E) Schematic of the principle of gene expression in Cx3cr1-Cre/Chil3-Flp/VeDTR (LF) mice (CCD mice). In CCD mice, DTR and YFP are expressed only in cells that express both Cx3cr1-Cre and Chil3-Flp. (F and G) The identification of YFP^+^ cells in macrophage of CCD mice. Flow cytometric analysis showing YFP positivity in lung cells (F) and macrophages of systemic organs (G) in C57BL/6 (gray, *n* = 3) and CCD mice (black, *n* = 3).

In the VeDTR mouse system, two different genes have to be selected to target specific cell types of interest.^36^ We selected *Cx3cr1* as the first gene to target AMs because Cx3cr1-Cre mice are widely used to label various tissue macrophages, including AMs.^37^ To test whether Cx3cr1-Cre mice can be used in the VeDTR system, we examined YFP expression in the lung myeloid cells of Cx3cr1-Cre/VeDTR (ΔFRT) mice (Figure S1A). In these mice, Cre expression alone allowed the VeDTR mice to express YFP and DTR because the FRT-flanked stop cassette was deleted. We found that YFP was detected in AMs as well as in other myeloid cells throughout the body in Cx3cr1-Cre/VeDTR (ΔFRT) mice (Figure S1B). To find the second gene to generate *Flp* transgenic mice, we next performed bulk RNA sequencing (bulk RNA-seq) and compared the gene expression profiles of YFP^+^ AMs with those of other YFP^+^ cells in the lung myeloid cells of Cx3cr1-Cre/VeDTR (ΔFRT) mice (Figures 1A–1C). On the basis of the database of gene expression profiles (BioGPS) and bulk RNA-seq results from previous studies,^38^ we selected 15 candidate genes that show high expression in the lungs and low expression in other organs (Figure 1B). Among these, the *Chil3* gene, which encodes the Ym1 protein that is mainly produced by M2 macrophages induced in *in vitro* culture,^39^ had the highest expression level in YFP^+^ AMs (Figure 1C). We also confirmed that *Chil3* mRNA was selectively expressed in AMs but not in other lung myeloid cells by quantitative RT-PCR (qPCR) (Figure 1D). Therefore, we chose *Chil3* as the second gene candidate in the VeDTR mouse system for targeting AMs (Figure 1E). We generated Chil3-Flp mice, in which the T2A-Flp recombinase cassette was inserted into the last amino acid codon of the *Chil3* gene, by genome editing (Figure S1C). In Cx3Cr1-Cre/Chil3-Flp/VeDTR (LF) mice (CCD mice), YFP and DTR expression should theoretically be allowed when cells have doubly expressed *Cx3Cr1* and *Chil3* genes in the VeDTR mouse system (Figure 1E). To analyze whether YFP is expressed selectively in AMs, we performed flow cytometry on lung myeloid cells and macrophages in systemic organs in C57BL/6 and CCD mice. Notably, YFP was detected only in AMs and not in various lung myeloid cells such as interstitial or inflammatory macrophages, dendritic cells, neutrophils, and eosinophils (Figures 1F and S2A). The YFP-positive rate of AMs in CCD mice appeared to be approximately 70% (Figure S2A). This lower-than-expected value (less than 100%) was likely due to the strong autofluorescence of AMs in WT mice, which made some cells appear YFP-positive and affected the ability to distinguish YFP-positive AMs in CCD mice. However, in the histogram (Figure 1F), the entire AM population is YFP-positive, and no bimodal distribution is observed, indicating that all AMs are YFP-positive in CCD mice. Furthermore, we did not detect any YFP-positive cells among various tissue-resident macrophages (Figures 1G and S2B) or the peripheral blood mononuclear cells (PBMCs) from the CCD mice (Figures S2C and S2D). When we compared the expression levels of *Chil3* (a well-known marker of M2 macrophages^39^) in AMs from wild-type mice with those in macrophages from other tissues using qPCR (Figure S2E), the expression level of *Chil3* in AMs was much higher than those not only in resident macrophages from the spleen, brain, and bone marrow but also in M2 macrophages induced by stimulating bone marrow derived macrophages (BMDMs) with IL-4 (Figures S2E and S2F). Although the IL-4 treatment itself increased the expression levels of *Chil3* and CD206 (Figures S2E and S2F), the induced M2 macrophages from CCD mice were YFP-negative. Collectively, these findings indicate that AMs are selectively labeled with YFP in CCD mice.

### Conditional AM depletion in CCD mice

**Figure 2:**
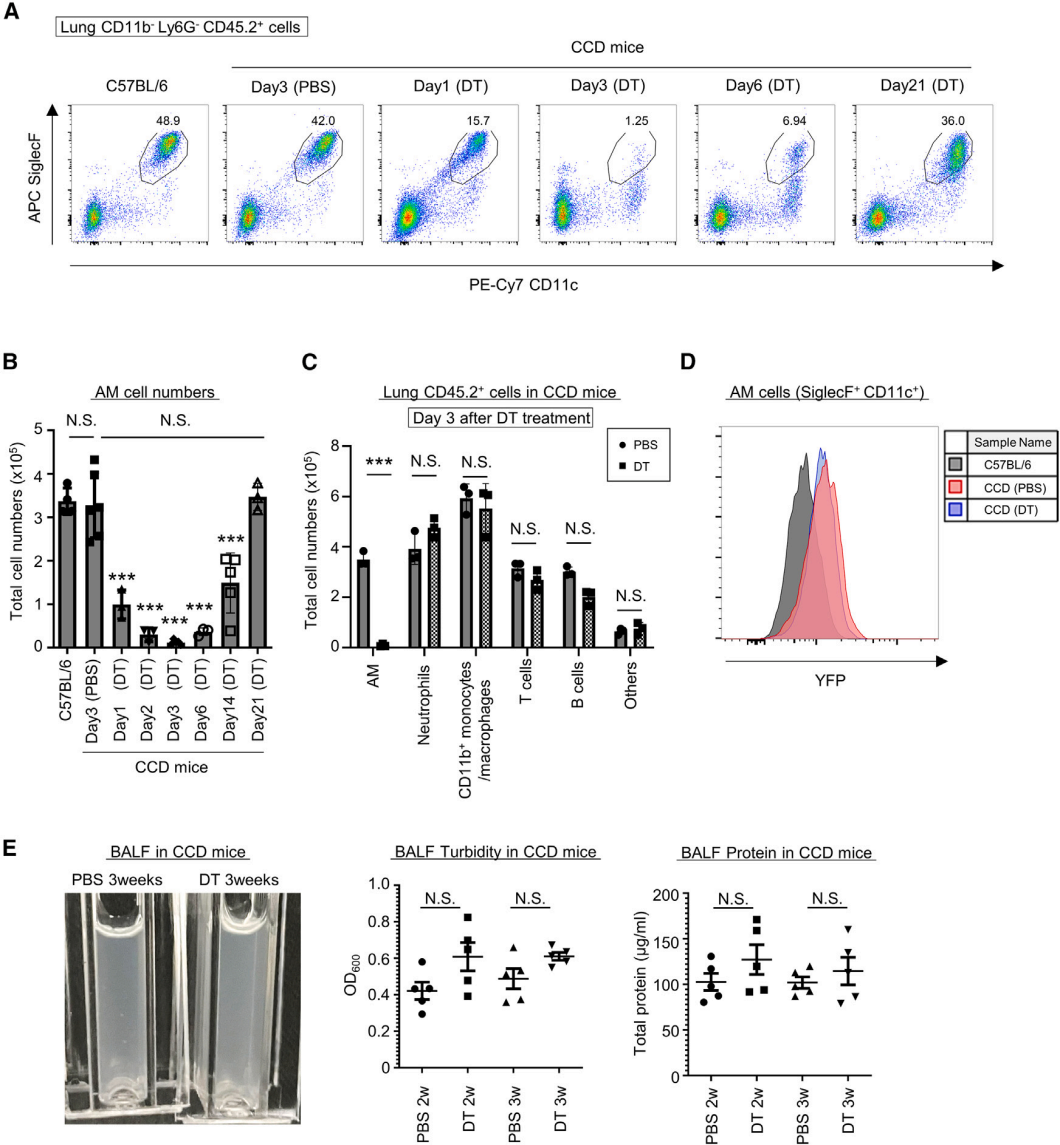
Selective depletion of AMs with DT in CCD mice (A and B) Flow cytometric analysis of AMs in the lungs on indicated days after single intraperitoneal administration of PBS or DT to WT and CCD mice (n = 3–6 each), with their plot diagram (A) and AM cell count (B). The black line in the plot diagram refers to AMs and the numbers indicate the percentage of AMs among CD11b^−^ Ly6G^−^ CD45.2^+^ cells. (C) Number of cells in the lungs of CCD mice on 3 days after PBS (circle) or DT (square) single administration (*n* = 3 each), including AMs (SiglecF^+^ CD11c^+^ CD11b^−^ Ly6G^−^), neutrophils (Ly6G^+^ CD11b^+^), CD11b^+^monocytes/macrophage (CD11b^+^ Ly6G^−^), T cells (CD3^+^ B220^−^), B cells (B220^+^ CD3^−^), others (SiglecF^−^ CD11c^−^ CD11b^−^ Ly6G^−^ CD3^−^ B220^−^ CD45.2^+^). (D) Analysis of YFP positivity AMs by flow cytometry in C57BL/6 (black) and CCD mice after 21 days of PBS (red) or DT (blue) administration (*n* = 3 each). (E) BALF turbidity and total protein amount in CCD mice treated with PBS or DT for 2 and 3 weeks (*n* = 5 each). The optical density of BALF was measured at 600 nm (OD_600_). Protein levels were estimated using a calibration curve based on absorbance at 540-490 nm with CBB staining. Data are representative of two independent experiments (A, D, E), and cumulative of two independent experiments (B and C). Statistical significance assessment: one-way ANOVA with Tukey’s multiple comparisons test (B and E) and two-way ANOVA with Sidak’s multiple comparisons test (C) Error bars represent SD. ∗∗∗*p* < 0.001, N.S.: not significant.

The AMs of CCD mice should express not only YFP but also DTR (Figure 1E), and therefore we next attempted to deplete AMs by injecting DT (Figures 2A and 2B). A single intraperitoneal injection of DT gradually depleted AMs in a time-dependent manner. Specifically, approximately 97% of AMs were depleted 3 days after a single injection of DT (Figures 2A and 2B). Flow cytometry on day 3 after DT treatment showed no change in the number of immune cells in the lungs except for AMs (Figure 2C). AMs alone were depleted on days 1.5 and 10 after DT treatment in CCD mice (Figures S3A and S3B), demonstrating AM-specific elimination in CCD mice. Next, we assessed whether AMs recovered after the DT injection. We detected the recovery of AMs on day 21 after DT injection, in terms of the cell surface markers and cell numbers (Figures 2A and 2B). Moreover, the recovered AMs expressed YFP (Figure 2D), suggesting the qualitative and quantitative recovery of AMs in CCD mice after the depletion. Constitutive AM depletion leads to pulmonary alveolar proteinosis.^17^ Therefore, we next examined whether CCD mice, upon the transient AM depletion, develop pulmonary alveolar proteinosis. When CCD mice were injected with DT or PBS every 2–3 days and kept AM-free for 2–3 weeks, there were no significant differences in bronchoalveolar lavage fluid (BALF) turbidity and total protein levels between the PBS- and DT-treated groups (Figure 2E). This suggested that, unlike constitutive AM depletion in GM-CSF-deficient mice, the transient AM depletion in CCD mice did not lead to the development of pulmonary alveolar proteinosis after the depletion of AMs for 3 weeks. In addition, although the depletion of AMs was sustained following continuous DT treatment for 2 weeks, we observed a slight but significant increase in the number of CD11b^+^ monocytes/macrophages (Figure S3C). Taken together, these results indicate that AMs can be selectively depleted in CCD mice in the short term without affecting the number of other cells or the accumulation of alveolar surfactant in the airways.

### AMs transiently prevent acute *M. abs* infection

**Figure 3:**
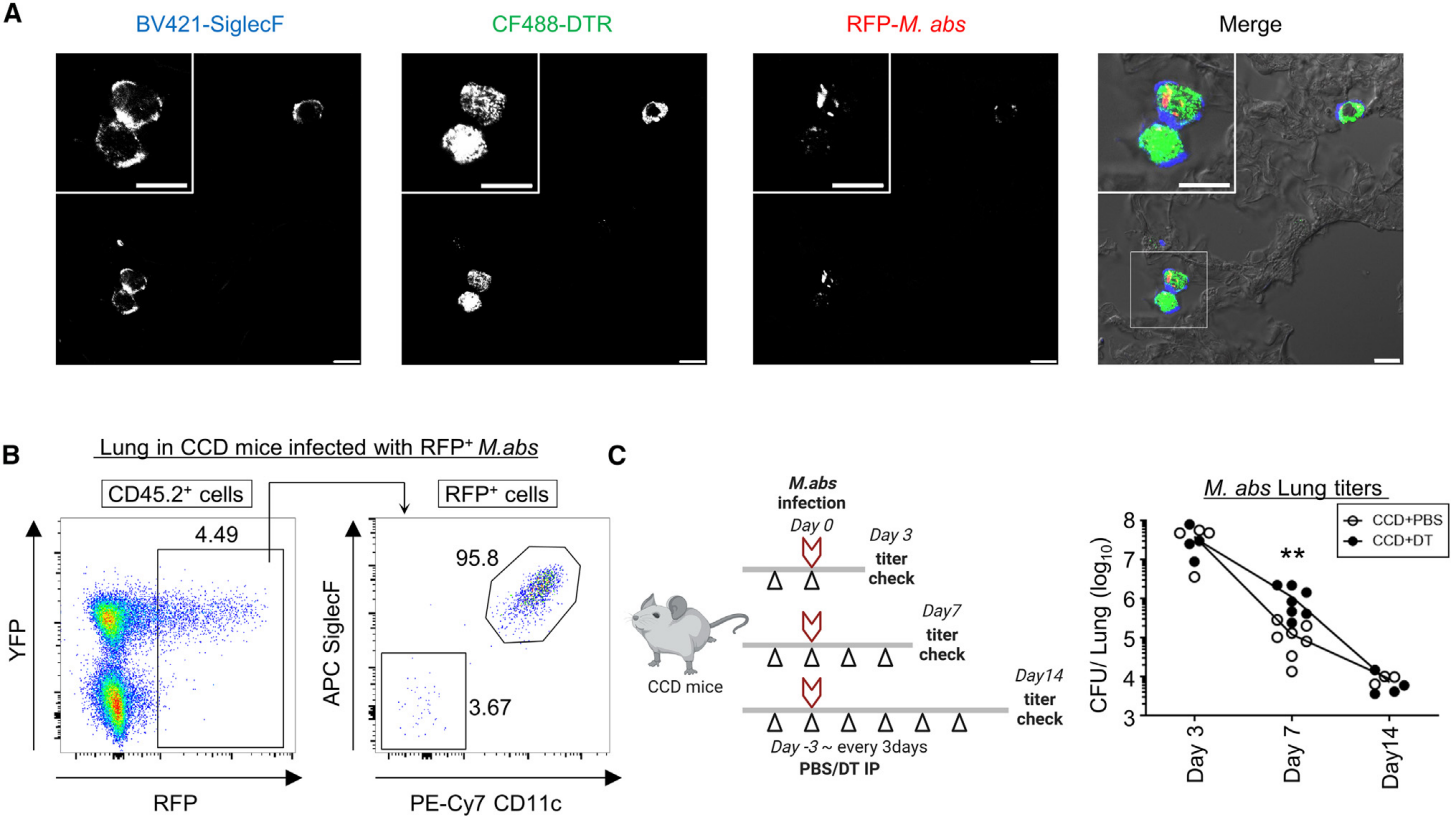
The role of AMs in *Mycobacterium abscessus* infections (A) Immunofluorescence microscopy of the lungs of CCD mice (*n* = 3) at 10 h after intranasal infection with RFP-expressing *Mycobacterium abscessus* (*M. abs*). The lung stained with anti-SiglecF and anti-DTR. The white square area is magnified in the upper left corner. All scale bars indicate 10μm. (B) Flow cytometric analysis of the lungs of infected CCD mice with RFP-expressing *M. abs* at 10 h after infection (*n* = 6). The numbers in the left plot diagram indicate the percentage of infected cells by RFP-expressing *M. abs* in CD45.2^+^ cells. The numbers in the right plot diagram indicate the percentage of SiglecF^+^CD11c^+^ AMs and others in RFP^+^ cells. (C) *M. abs* bacterial load on indicated days of intranasal infection in the lungs of CCD mice pretreated with PBS (white) or DT (black) (n = 3–7 each). The diagram on the left shows the experimental schedule. The black and red arrows in the diagram indicate the time of PBS or DT treatment and infection, respectively. Data are representative of two independent experiments. Statistical significance assessment: two-way ANOVA with Sidak’s multiple comparisons test (C) and unpaired two-tailed Student’s *t* test (D and E). Error bars represent SD. ∗*p* < 0.05, ∗∗*p* < 0.01, ∗∗∗*p* < 0.001.

It has been shown that *Mycobacterium* spp. first infect AMs during respiratory infection.^26^ To analyze the role of AMs in mycobacterial infection, we first assessed whether mCherry (RFP)-expressing *M. abs* infects AMs in CCD mice using immunohistochemistry and flow cytometry (Figures 3A and 3B). Immunohistochemistry of lung sections from the infected CCD mice showed that RFP was detected in cells visualized with both DTR and SiglecF (Figure 3A). Moreover, at 10 h after intranasal infection with RFP-expressing *M. abs* in CCD mice, almost all RFP^+^ populations were YFP^+^ cells, suggesting the highly selective infection of *M. abs* in AMs (Figure 3B). To examine whether *M. abs* infection affects the expression of YFP and DTR in CCD mice, we performed flow cytometry on the lungs and PBMCs of CCD mice on day 7 after infection with *M. abs*. We did not detect YFP in any cell types tested, except for AMs (Figures S4A–S4D). Because AMs are immune cells that initially encounter *M. abs* during respiratory infection, we investigated whether AMs promote or inhibit *M. abs* infection. The bacterial loads in the lungs were assessed on days 3, 7, and 14 after intranasal infection with *M. abs* in CCD mice pretreated with DT or PBS. Although the titers on day 3 after infection were comparable between the two groups, it was noteworthy that the *M. abs* titers in DT-treated mice were 9-fold higher than those in PBS-treated mice on day 7 after infection (Figure 3C). Meanwhile, the bacterial titers on day 14 after infection decreased compared with those on day 7 and were comparable in the presence or absence of AMs (Figure 3C). This indicated that AMs may transiently inhibit *M. abs* proliferation in the lungs in the early stages of infection.

We next examined the role of AMs for other typical human pathogens involved in respiratory infection, namely, *S. pneumoniae* (*Spn*) and SARS-CoV-2. A previous study showed that AM depletion by administering clodronate liposomes led to an increase in *Spn* load and the exacerbation of pneumonia, suggesting that AMs prevent *Spn* replication in the lungs.^40^^,^^41^ To test this, we infected CCD mice with *Spn* and measured the titers in the lungs. Surprisingly, the *Spn* titers in the DT-treated group were one-eighth of those in the PBS-treated group (Figure S5A), suggesting that AMs facilitate *Spn* replication in the lungs. A previous study showed that AMs promoted SARS-CoV-2 replication in the lungs of hamsters administered clodronate liposomes, subsequently intranasally infected with SARS-CoV-2, and then tested for viral titers.^42^ To test this, we generated a C57BL/6 mouse-adapted SARS-CoV-2 strain to infect CCD mice and measured the viral titers in the lungs (Figure S5B). In contrast to the previous results, AM depletion in CCD mice led to an increase in SARS-CoV-2 titers in the lungs, suggesting that AMs prevent SARS-CoV-2 replication there. In summary, the removal of AMs using the VeDTR mouse system revealed a protective role of AMs against *M. abs* and SARS-CoV-2, but a growth-supporting role for *Spn*.

### IFN-γ-dependent AM disappearance contributes to reducing bacterial titer in *M. abs* infection

**Figure 4:**
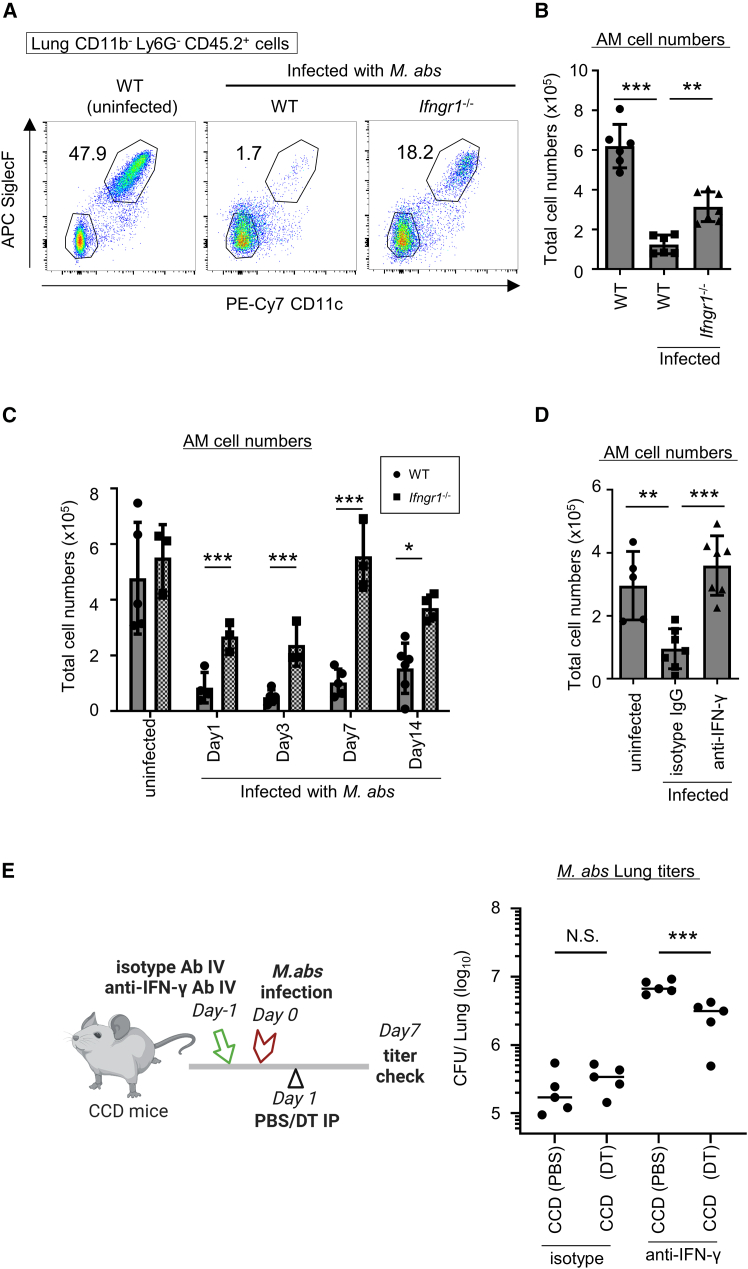
IFN-γ-dependent AM disappearance contributes to *M. abs* reduction (A and B) Flow cytometric analysis of AMs in the lungs of WT or *Ifngr1*^−/−^ mice (*n* = 6 each) on 7 days after *M. ab*s intranasal infection with their plot diagram (A) and AM cell count (B). The numbers in the plot diagram indicate the percentage of AMs among CD11b^−^ Ly6G^−^ CD45.2^+^ cells. (C) The number of AM cells in the lungs of WT (circle) or *Ifngr1*^−/−^ (square) mice (n = 3–6 each) on indicated days after intranasal infection with *M. abs.* (D) The number of AM cells in the lungs of WT mice on 7 days after *M. abs* infection. The mice were pre-treated intravenously with isotype-control or anti-IFN-γ antibody 24 h before infection (n = 5–8 each). (E) *M. abs* bacterial load on day 7 of infection in the lungs of CCD mice (*n* = 5 each). The CCD mice were treated intravenously with either isotype-control or anti-IFN-γ antibody 24 h prior to infection and injected with either PBS or DT one day after infection. The diagram on the left shows the experimental schedule. The black, red and green arrows in the diagram indicate the time of DT treatment, infection and antibody treatment, respectively. Data are representative of two independent experiments. Statistical significance assessment: one-way ANOVA with Tukey’s multiple comparisons test (B, E and F), two-way ANOVA with Sidak’s multiple comparisons test (C) and unpaired two-tailed Student’s *t* test (D). Error bars represent SD. ∗*p* < 0.05, ∗∗*p* < 0.01, ∗∗∗*p* < 0.001, N.S.: not significant.

AM disappearance has been shown to occur during infection of the respiratory tract by microbes.^11^ We also found that AMs disappeared from the lungs of C57BL/6 WT mice on day 7 after intranasal *M. abs* infection (Figures 4A and 4B). A recent study also demonstrated that the disappearance of peritoneal tissue-resident macrophages during *Toxoplasma gondii* infection involves IFN-γ-dependent cell death.^43^ To build on this previous work, we examined whether IFN-γ is involved in *M. abs*-induced AM disappearance. We found that AM disappearance in IFN-γ receptor-deficient mice (*Ifngr1*^−/−^ mice) intranasally infected with *M. abs* was not as profound as that in WT mice (Figures 4A and 4B). Next, we investigated for how long the disappearance of AMs persisted in *M. abs*-infected WT mice. Time course analysis showed that the number of AM cells in the lungs decreased on day 1 after infection and did not recover to the uninfected level until at least 2 weeks later (Figure 4C). In sharp contrast, although AM cell numbers in *M. abs*-infected *Ifngr1*^−/−^ mice transiently decreased at early timepoints after infection, they recovered at later timepoints (Figure 4C). Intravenous administration of anti-IFN-γ antibodies also prevented the *M. abs* infection-induced disappearance of AMs (Figure 4D), demonstrating that IFN-γ is involved in AM disappearance during *M. abs* infection.

The above findings present a conundrum given that AMs were shown to be important for host defense against *M. abs* infection (Figure 3C). They raise the question of whether the IFN-γ-induced disappearance of AMs during infection really plays a role in preventing *M. abs* infection. To answer this question, we attempted to artificially reproduce the disappearance of AMs during infection by administering DT to the CCD mice because previous studies demonstrated that both DT-dependent cell death and IFN-γ-induced AM disappearance depend on pyroptosis or apoptosis.^44^^,^^45^^,^^46^^,^^47^^,^^48^^,^^49^ After the administration of isotype or anti-IFN-γ antibody, mice were infected with *M. abs*, and DT or PBS was administered to artificially induce AM disappearance the next day (Figure 4E). In the group administered isotype antibody, AM disappeared due to infection regardless of the presence or absence of DT, and the bacterial loads were comparable between PBS- and DT-treated groups. Meanwhile, in the anti-IFN-γ antibody-administered CCD mice, AM disappearance was inhibited, and the bacterial loads were markedly increased compared with those in the mice administered isotype control antibody. By contrast, in the anti-IFN-γ-administered CCD mice, the bacterial loads in the DT-treated groups were significantly lower than those in the PBS-treated groups (Figure 4E), suggesting that the IFN-γ-induced AM disappearance might suppress *M. abs* proliferation. On the basis of these results (Figures 3C and 4E), AMs might prepare to confront *M. abs* invasion before infection and, following infection, rapidly disappear along with infected cells in an IFN-γ-dependent manner, eventually suppressing *in vivo M. abs* proliferation during infection. However, because DT treatment could not completely prevent the increased bacterial load in IFN-γ antibody-treated mice (Figure 4E), mechanisms other than AM disappearance are required for IFN-γ to protect against *M. abs* infection. Taken together, these findings suggest that IFN-γ-induced AM disappearance may be required for *M. abs* clearance.

### AMs are essential for GM-CSF-mediated defense against *M. abs* infection

**Figure 5:**
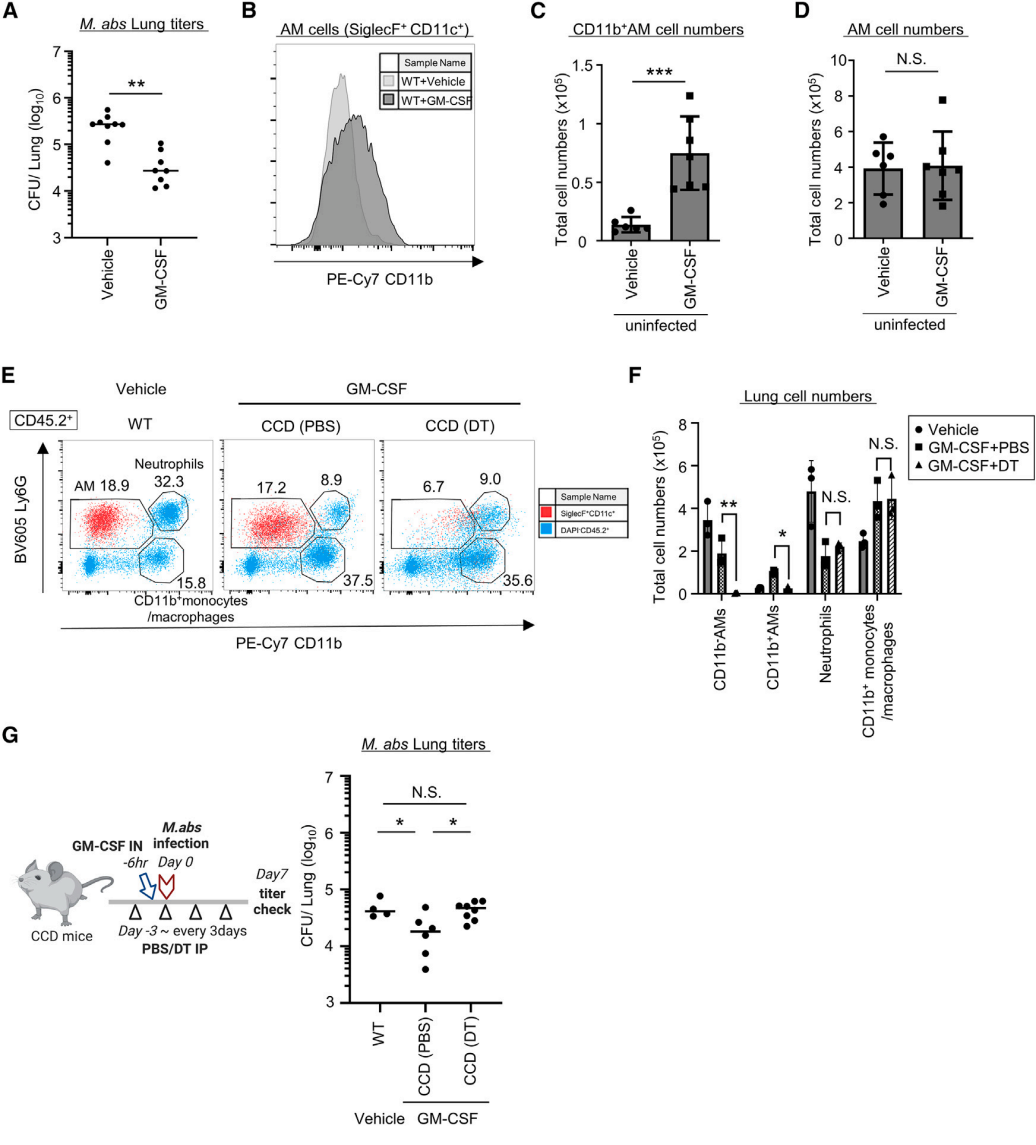
GM-CSF inhalation therapy acts directly on AMs to reduce *M. abs* (A) *M. abs* bacterial load on day 7 of infection in the lungs of WT mice that were pre-treated intranasally with vehicle (0.1% BSA) or recombinant mouse GM-CSF (n = 8–9 each) at 6 h prior to infection. (B–D) Flow cytometric analysis of the lungs of AMs in WT mice that were treated intranasally with vehicle or recombinant mouse GM-CSF (n = 6–7 each) after 2 days, with their CD11b positivity (B), CD11b^high^ AM cell count (C) and total AM cell count (D). (E and F) Flow cytometric analysis of the lungs of WT and CCD mice (*n* = 3 each) on 2 days after treatment with either vehicle or GM-CSF inhalation with their plot diagram (E) and lung cell count (F). WT mice were treated with vehicles (circle in F). CCD mice were pretreated with PBS (square in F) or DT (triangle in F) on day −3 and day 0, followed by intranasal GM-CSF treatment. The plots show pre-gated live immune cells (DAPI^−^ CD45.2^+^; blue in E) and AMs (SiglecF^+^ CD11c^+^ DAPI^−^ CD45.2^+^; red in E), then expanded with Ly6G and CD11b gated with AMs (SiglecF^+^ CD11c^+^ CD11b^−^ Ly6G^−^), neutrophil (Ly6G^+^ CD11b^+^), CD11b^+^ Mo/MΦ (CD11b^+^ Ly6G^−^). The numbers in the plot diagram indicate the percentage of AMs, neutrophils and CD11b^+^monocytes/macrophages among CD45.2^+^ cells. (G) *M. abs* bacterial load on day 7 of infection in the lungs of WT mice and CCD mice (n = 4–8 each). The WT mice were pre-treated with vehicle 6 h prior to infection. The CCD mice were pretreated with PBS or DT, then intranasally administered GM-CSF, and infected with *M. abs* 6 h later. Data are representative of two independent experiments. Statistical significance assessment: unpaired two-tailed Student’s *t* test (A, C, and D) and one-way ANOVA with Tukey’s multiple comparisons test (F). Error bars represent SD. ∗*p* < 0.05, ∗∗*p* < 0.01, ∗∗∗*p* < 0.001, N.S.: not significant.

Exogenous GM-CSF administration is reported to effectively reduce *Mycobacterium* titers in mice and human patients.^33^^,^^34^^,^^35^^,^^50^^,^^51^ Because GM-CSF is an essential growth factor for AM maturation,^16^ it is possible that the efficacy of GM-CSF inhalation depends on AMs, but the causal relationship between them remains unclear. To test this possibility, we first attempted to establish a mouse model of GM-CSF inhalation therapy against *M. abs* infection in accordance with previous experiments in which recombinant GM-CSF was administered.^52^ Recombinant GM-CSF or control was administered intranasally 6 h prior to *M. abs* infection, and the bacterial loads were assessed on day 7 after infection (Figure 5A). The bacterial loads of *M. abs* in the lungs of GM-CSF-treated mice were one-seventh of those in control mice (Figure 5A), indicating that the intranasal inhalation of recombinant GM-CSF protein effectively reduces the number of *M. abs* in the lungs. Next, we assessed how AMs were affected by GM-CSF administration using flow cytometry (Figure 5B). We found that surface CD11b levels on AMs were increased after the GM-CSF inhalation, albeit weakly in AMs from the control mice (Figure 5B). The numbers of CD11b^high^ AMs significantly increased (Figure 5C) while the total number of AMs was comparable between the GM-CSF-inhaled and control groups on day 2 after treatment (Figure 5D). To determine whether the efficacy of GM-CSF treatment depends on AMs, CCD mice pretreated with PBS or DT were administered GM-CSF (Figures 5E and 5F), followed by *M. abs* infection (Figure 5G). Flow cytometric analysis showed that the AMs (SiglecF^+^ CD11c^+^) in which surface expression of CD11b increased after GM-CSF treatment specifically disappeared upon DT treatment in CCD mice (Figures 5E and 5F). By contrast, neutrophils (CD11b^high^ Ly6G^+^) and the CD11b^+^ monocyte/macrophage population (CD11b^high^ Ly6G^−^) showed no differences in number between the PBS- and DT-treated groups after GM-CSF stimulation (Figure 5F). This suggests that the population of CD11b^+^ SiglecF^+^ CD11c^+^ cells whose abundance is increased by GM-CSF is derived from AMs. We found that the bacterial titers of *M. abs* in the lungs of the DT-treated group were significantly higher than those of the PBS-treated group, despite GM-CSF treatment (Figure 5G). In addition, the bacterial titers after AM depletion were comparable to those in vehicle-treated mice (Figure 5G). Although GM-CSF treatment changed the numbers of neutrophils and CD11b^+^ monocytes/macrophages compared with those upon vehicle treatment (Figure 5F), the results in Figure 5G clearly indicate that GM-CSF treatment mainly targets AMs in terms of its therapeutic effect on *M. abs*.

Finally, we investigated how GM-CSF treatment affects AM disappearance during infection. Wild-type mice were infected with *M. abs* 6 h after GM-CSF or vehicle treatment, and flow cytometry was performed on day 7 after infection (Figures S6A and S6B). Regardless of whether or not GM-CSF treatment was performed, the AMs disappeared, while no significant effects were observed on other lung cell populations. Taken together, these results suggest that AMs are indispensable for GM-CSF inhalation-mediated defense against *M. abs* infection.

## Discussion

VeDTR mouse technology can be applied to all cell types using two distinct genes.^36^ In this study, we chose *Cx3cr1* and *Chil3*, and succeeded in generating a unique mouse line in which AMs are specifically labeled by YFP and can be depleted by DT (Figure 1E). AMs do not actually express *Cx3cr1*, but Cx3cr1-Cre has previously been expressed in yolk sac-derived AM precursors,^37^ and thus AMs could be labeled in CCD mice. To further establish mice with more strictly time-specific conditional KO, the Cre-ERT2 system to control *Cre* expression only upon tamoxifen administration can be used. *Chil3* is an M2 macrophage marker that is induced by IL-4 and/or IL-13 stimulation *in vitro.*^53^ *Chil3* is expressed in the induced M2 macrophages and other macrophages in CCD mice, but its expression in their macrophages was much lower than in AMs (Figures S2E and S2F). This suggests that the low levels of *Chil3* expression in the induced M2 macrophages may be insufficient for *Flp* recombinase-mediated excision of the *FRT* cassette in the VeDTR mouse system. The recombination efficiency of *Flp-FRT* is lower than that of *Cre*-*loxP*,^54^ which may have allowed the cell specificity of AMs in CCD mice. In the VeDTR mouse model, the second gene controlling *Flp* recombinase expression must be carefully selected in terms of the expression level as well as the specificity.

We have also shown that AMs play a role in the suppression of *M. abs* and SARS-CoV-2 proliferation (Figures 3C and S5B). The results in *M. abs* are also consistent with the epidemiology of susceptibility to mycobacterial infection in human patients with pulmonary alveolar proteinosis.^55^ In contrast, *Spn* titers were lower when AMs were removed from CCD mice (Figure S5A). These results contrast with previous studies in which clodronate liposomes were used to deplete macrophages.^14^^,^^40^^,^^42^ The increase in bacterial load upon AM depletion with clodronate liposomes in pneumococcal infection may be due to the low cell selectivity of clodronate liposomes, which also impair neutrophil function.^15^ Pneumococcal infection was shown to be more severe when neutrophils were eliminated with anti-Ly6G antibody,^56^ and when mice with deficiency in cytokines promoting neutrophil migration were used.^57^ Furthermore, AMs may regulate inflammation and delay the migration of neutrophils and other phagocytic cells through immune tolerance. Alternatively, AMs could serve as a reservoir for intracellular *Spn*, helping to control bacterial load during infection. However, the exact mechanisms involved remain unclear. Regarding the SARS-CoV-2 response, our study found that depletion of AMs led to an increase in SARS-CoV-2 titers. In contrast, a previous study reported that AM depletion using clodronate liposomes in hamsters resulted in a decrease in viral titers.^42^ The reason for this discrepancy remains unclear. This might have been due to the distinct methods used and/or the different animals, viral strains, or detection methods.

Our results revealed the significance of IFN-γ-dependent AM disappearance. Some reports have described that infection-induced AM disappearance is associated with caspase-1-dependent pyroptosis and TNFα-dependent apoptosis.^46^^,^^47^^,^^48^^,^^49^ Additionally, IFN-γ-primed BMDMs can induce caspase-8-dependent apoptosis through TLR4 stimulation.^58^ It is possible that our IFN-γ-dependent AM disappearance captures the same phenomenon as in these studies. Even if IFN-γ-dependent AM disappearance was defective, the artificial AM disappearance during the acute phase of infection reduced the number of *M. abs* in the lungs. These results suggest that AM disappearance itself plays a role in the suppression of mycobacterial proliferation. This disappearance may also contribute to host defense by sequestering intracellular *M. abs* and losing host cells infected by the bacteria.

In the present study, we demonstrated that the efficacy of GM-CSF therapy against *M. abs* significantly decreased when AMs were removed, strongly indicating that it requires AMs to exert its effects (Figures 5A and 5G). GM-CSF inhalation therapy itself has already been established as an effective and safe treatment for patients with autoimmune pulmonary alveolar proteinosis.^59^ Recently, an open-label, comparative cohort study also reported that GM-CSF inhalation may be effective in treating refractory NTM infections.^33^ However, the reason why GM-CSF was effective remains unknown; studies in 2013 and 2018 demonstrated that adenoviral vectors expressing GM-CSF effectively reduced *Mtb* and *M. bovis* BCG titers.^50^^,^^51^ This was shown to be because GM-CSF activates type I immune responses in macrophages via IRF5 expression,^60^^,^^61^ and GM-CSF-treated AMs also exhibited increases in the expression of iNOS and inflammatory cytokines.^50^^,^^51^^,^^52^ We found that the GM-CSF inhalation upregulated CD11b in AMs. Given that CD11b^high^ AMs have been reported to express higher levels of inflammatory macrophage markers and cytokines than their CD11b^low^ counterparts,^62^ the GM-CSF treatment might have activated the AMs. We found that GM-CSF-induced CD11b^+^ AMs eventually disappeared during infection (Figures S6A and S6B). Given that CD11b is a receptor of complement and plays a role in pathogen clearance,^63^ it is possible that GM-CSF-induced CD11b^+^ AMs exhibit higher phagocytic and bactericidal activity or disappear more rapidly than CD11b^−^ ones. However, it remains unknown whether CD11b^high^ AMs have higher bactericidal capacity for *M. abs* than CD11b^low^ ones. The mechanism by which AMs prevent the proliferation of *M. abs* should be examined in future work. Taking the obtained findings together, considering that GM-CSF inhalation could become the new standard of care for refractory mycobacterial infections, our study reveals an indispensable role of AMs in inhaled GM-CSF’s effects against NTM.

In summary, this AM-specific depletion system in mice has provided us with new insights into the significance of AMs during *M. abs* infections. Hereafter, the usage of CCD mice would allow us to study the detailed roles of AMs in a wide range of respiratory infections and various disease models from a different perspective in a more specific way than previous methods.

### Limitations of the study

The CCD mice in this study have several limitations. First, while YFP expression in CCD mice was specific to AMs both in uninfected conditions and during *M. abs* infection (Figures 1F, 1G, and S4A–S4D), it is unclear whether the specificity to AMs would be maintained under other conditions, such as in models of allergic pulmonary diseases. Second, the depletion of AMs using DT in CCD mice is challenging to sustain over an extended period. Our previous study demonstrated that continuous administration of DT to VeDTR mice leads to the production of anti-DT antibodies in the mice by 3-4 weeks, preventing the depletion of target cells.^36^ Furthermore, when DT was administered continuously to CCD mice for 2 weeks, CD11b^+^ monocytes and macrophages infiltrated the lungs (Figure S3C), indicating that short-term depletion of AMs is preferable in CCD mice. Third, this study revealed that IFN-γ-dependent AM disappearance and GM-CSF-treated AM suppress *M. abs* infection (Figures 4E and 5G). However, the precise mechanism by which AM prevents infection remains unclear. Additionally, further studies are required to clarify the behavior of AM during pneumococcal and SARS-CoV-2 infections.

## Resource availability

### Lead contact

Further information and requests for resources and reagents should be directed to and will be fulfilled by the lead contact, Masahiro Yamamoto (myamamoto@biken.osaka-u.ac.jp).

### Materials availability

The CCD mouse strain, the transgenic *M. abscessus* strains, and unique reagents generated in this study are available upon request from the lead contact.

### Data and code availability

• Data: The published article and supplemental information include all data generated and analyzed during this study. RNA-seq data have been deposited at GEO and are publicly available as of the date of publication. Accession numbers are listed in the key resources table.
• Code: This paper does not report original code.
• Additional information: Any additional information required to reanalyze the data reported in this paper is available from the lead contact upon request.

## Acknowledgments

We thank Mari Enomoto and Nodoka Yamagishi (Osaka University) for secretarial assistance. This study was supported by 10.13039/501100002241Japan Science and Technology Agency (JPMJFR206D and JPMJMS2025); Agency for Medical Research and Development (JP24fk0108682, JP223fa627002, and JP24fk0108673); 10.13039/501100001700Ministry of Education, Culture, Sports, Science and Technology (24K22056); the program from Joint Usage and Joint Research Programs of the Institute of Advanced Medical Sciences, 10.13039/501100005623Tokushima University; 10.13039/100007449Takeda Science Foundation; Mochida Memorial Foundation; 10.13039/501100007263Astellas Foundation for Research on Metabolic Disorders; 10.13039/100007428Naito Foundation; the 10.13039/100024224Chemo-Sero-Therapeutic Research Institute; 10.13039/501100019670Research Foundation for Microbial Diseases of Osaka University; BIKEN Taniguchi Scholarship; The Nippon Foundation - Osaka University Project for Infectious Disease Prevention; Joint Research Program of Research Center for Global and Local Infectious Diseases of 10.13039/501100007510Oita University (2021B06); and the Research Fellow of Scholarship for Doctoral Students in Immunology.

## Author contributions

Conceptualization, Y.N., M.S., and M.Y.; methodology, Y.N., A.K., M.O., and M.Y.; formal analysis, Y.N., D.O., M.S., and M.Y.; investigation, Y.N., M.S., and M.Y.; resources, K.Y., C.O., M.Y., S.K., N.S., Y.O., Y.M., and M.A.; writing – original draft, Y.N. and M.Y.; writing – review and editing, Y.N., M.S., and M.Y.; funding acquisition, M.Y.; supervision, M.Y.

## Declaration of interests

The authors declare no conflict of interest.

## STAR★Methods

### Key resources table

**Table:** 

REAGENT or RESOURCE	SOURCE	IDENTIFIER
**Antibodies**		

Purified anti-mouse CD16/32 (93)	Biolegend	Cat#101302; RRID:AB_312801
APC anti-mouse CD170 (Siglec-F)	Biolegend	Cat#155508; RRID:AB_2750236
APC-Cy7 anti-mouse CD45.2 (104)	Biolegend	Cat#109823; RRID:AB_830788
APC-Cy7 anti-mouse/human CD11b (M1/70)	Biolegend	Cat#101226; RRID:AB_830641
APC-Cy7 anti-mouse/human CD45R/B220(RA3-6B2)	Biolegend	Cat#103224; RRID:AB_313006
APC-Cy7 anti-mouse F4/80(QA17A29)	Biolegend	Cat#157315; RRID:AB_2894638
BV605 anti-mouse Ly-6G (1A8)	Biolegend	Cat#127639; RRID:AB_2565880
PE anti-mouse CD206 (MMR) Antibody (C068C2)	Biolegend	Cat# 141705; RRID:AB_10896421
PE anti-mouse CD3 Antibody(17A2)	Biolegend	Cat#100206; RRID:AB_312662
PE anti-mouse Ly-6C Antibody(HK1.4)	Biolegend	Cat#128008; RRID:AB_1186132
PE-Cy7 anti-mouse CD11b (M1/70)	Biolegend	Cat#101216; RRID:AB_312798
PE-Cy7 anti-mouse CD11c (N418)	Biolegend	Cat#117318; RRID:AB_493568
PE/Dazzle594 anti-mouse CD45.2 (104)	Biolegend	Cat#109846; RRID:AB_2564176
PE/Dazzle594 anti-mouse F4/80	Biolegend	Cat#123146; RRID:AB_2564133
PerCP-Cy5.5 anti-mouse CD11c (N418)	Biolegend	Cat#117328; RRID:AB_2129642
PerCP-Cy5.5 anti-mouseLy-6G/Ly-6C (RB6-8C5)	Biolegend	Cat#108410; RRID:AB_313374
APC anti-mouse F4/80 (BM8)	Biolegend	Cat#123116; RRID:AB_893481
APC-Cy7 anti-mouse CD19 (6D5)	Biolegend	Cat#115529; RRID:AB_830706
APC/Cyanine7 anti-mouse Ly-6G	Biolegend	Cat#127624; RRID:AB_10645331
PE anti-mouse CD64 (FcγRI) Antibody (X54-5/7.1)	BioLegend	Cat#139304; RRID:AB_10612740
PerCP-Cy5.5 anti-mouse I-A/I-E (M5/114.15.2)	BD	Cat#562363; RRID:AB_11153297
PerCP-Cy5.5 mouse anti-mouse CD45.2 (104)	BioLegend	Cat#109828; RRID:AB_893350
BV421 rat anti-mouse SiglecF	BD	Cat#562681; RRID:AB_2722581
Goat Anti-Human Hb-egf (DTR) antibody	R&D system	Cat#AF-259-NA; RRID:AB_354429
CF488A Donkey anti-goat IgG H&L	Biotium	Cat#20016; RRID:AB_10563028
Rat anti-IFN gamma *in vivo* (XMG1.2)	Bio X cell	Cat#BE0055; RRID:AB_1107694
Rat anti-horseradish peroxidase rat IgG isotype control *in vivo* (HRPN)	Bio X cell	Cat#BE0088; RRID:AB_110777

**Bacterial and virus strains**		

*Mycobacterium abscessuss* (ATCC19977)	Yoshida et al.^64^	N/A
mCheery expressing *Mycobacterium abscessuss* (ATCC19977)	This study	N/A
*Streptcoccuss pneumoniae* (TIGR4)	Yamaguchi et al.^65^	N/A
SARS-CoV-2 (B6 adapted)	This study	N/A

**Chemicals, peptides, and recombinant proteins**		

Ketamine	Daiichi Sankyo Propharma	Cat#1119400A3026
Xylazine	Elanco Japan	N/A
Isoflurane	Fujifilm	Cat#099-06571
Diphtheria Toxin	Millipore	Cat#322326
Bovine Serum Albumin	Sigma-Aldrich	Cat#A8806
Recombinant Murine GM-CSF	PeproTech	Cat#315-03
Collagenase D	Roche	Cat#11088882001
Dispase II	Roche	Cat#4942078001
Dispase II	gibco	Cat#17105041
DNase I	Roche	Cat#11284932001
Percoll	Sigma-Aldrich	Cat#P1644
HBSS (10x)	Gibco	Cat#14185-052
0.5mol/l EDTA (ph 8.0)	Nacalai Tesque	Cat#06894-14
sodium azide	Wako	Cat#195-11092
Trypan Blue Solution	Nacalai Tesque	Cat#20577-34
Formaldehyde Solution	Nacalai Tesque	Cat#16223-55
FSC22 Frozen Section Media	Leica	Cat#3801480
Tritonx-100	Nacalai Tesque	Cat#25987-25
Tween20	Nacalai Tesque	Cat#28353-85
Aqueous Mounting Medium, PermaFluor	Fisher Scientific	Cat#TA-030-FM
DAPI	Nacalai Tesque	Cat#11034-56
RPMI 1640 with L-Gln, liquid	Nacalai Tesque	Cat#30264-56
fetal bovine serum	Gibco	Cat#10270-106
2-mercaptoethanol	Nacalai Tesque	Cat#21417-52
Penicillin-Streptomycin Mixed Solution	Nacalai Tesque	Cat#26253-84
IL-4, Murine, Recombinant	PeproTech	Cat#214-14-20UG
BD DIFCO™ Middlebrook 7H9 Broth	BD	Cat#271310
Tube Middlebrook OADC Enrich 20ml	BD	Cat#211886
Glycerol	Nacalai Tesque	Cat#17017-35
Agar powder	Nacalai Tesque	Cat#01028-85
Todd-Hewitt broth	Millipore	Cat#T1438
Extract Yeast Dried	Nacalai Tesque	Cat#15838-45
TSAII 5% sheep blood agar	BD	Cat#251239

**Critical commercial assays**		

RNeasy Mini Kit	Qiagen	Cat#74106
Verso cDNA syntehsis Kit	Thermo Scientific	Cat#AB1453B
GoTaq qPCR Master Mix	Promega	Cat#A6002
Protein Assay CBB Solution (5-fold)	Nacalai Tesque	Cat#29449-44
KOD FX NEO	Toyobo	Cat#KFX-201
MEGAshortscript T7	Thermo Scientific	Cat#AM1354
mMESSAGE mMACHINE T7 ULTRA kit	Thermo Scientific	Cat#AM1345
MEGAclear kit	Thermo Scientific	Cat#AM1908

**Deposited data**		

Raw RNA sequencing data	This study	GEO: GSE271924

**Experimental models: Cell lines**		

Vero C1008 (Vero-E6)	ATCC	RRID: CVCL_0574
NCTC clone 929 (L-929)	ATCC	RRID: CVCL_0462

**Experimental models: Organisms/strains**		

C57BL/6N	SLC	N/A
*Ifngr1*-/-	Sasai et al.^66^	N/A
Cx3cr1-Cre	Kuratani et al.^67^	N/A
Chil3-Flp	This study	N/A
VeDTR (LF)	Okamoto et al.^36^	N/A
VeDTR (ΔFRT)	Okamoto et al.^36^	N/A

**Oligonucleotides**		

mBactin-Forward for qPCR primer: TTTGCAGCTCCTTCGTTGC	FASMAC	N/A
mBactin-Reverse for qPCR primer: TCGTCATCCATGGCGAACT	FASMAC	N/A
mChil3-Forward for qPCR primer: GTACCCTGGGTCTCGAGGAA	FASMAC	N/A
mChil3-Reverse for qPCR: CCTTGGAATGTCTTTCTCCACACG	FASMAC	N/A
Chil3-primer for generation of guide RNA	FASMAC	See STAR Methods in text
Chil3-primer for cloning	FASMAC	See STAR Methods in text

**Software and algorithms**		

FlowJo ver10.10	BD	N/A
GraphPad Prism 10.2.3	GraphPad	N/A
BioRender	BioRender	N/A
iDEP.96	iDEP.96 (Jul.2022)	http://bioinformatics.sdstate.edu/idep96/
BioGPS	BioGPS (Jul.2022)	http://biogps.org/

**Other**		

PT1300D homogenizer	Kinematica AG	N/A
Attune NxT flow cytometer	Thermo Scientific	N/A
FACS Aria III	BD	N/A
CFX connerct	Bio-Rad	N/A
CM1860 UV	Leica	N/A
FV3000	Olympus	N/A
NanoDrop-2000C	Thermo Scientific	N/A
iMark Microplate Absorbance Reader	Bio-Rad	N/A
T100 Thermal Cycler	Bio-Rad	N/A

### Experimental model and study participant details

#### Mice

C57BL/6NCrSlc mice were purchased from Japan SLC, Inc. *Ifngr1*^−^^/^^−^ mice, VeDTR mice and Cx3cr1-Cre mice have been previously described.^36^^,^^66^^,^^67^ All mice were maintained under specific pathogen free conditions. All experiments were performed on 6-16-week-old mice. Mouse experiments were randomized in both sexes. When the lungs of mice were collected, all mice were euthanized by inhalation of isoflurane (Fujifilm). All animal experiments were conducted with the approval of the Animal Research Committee of Research Institute for Microbial Diseases in Osaka University. Infection of mice with mouse-adapted SARS-CoV-2 virus were performed under the Animal Biosafety Level 3 (ABSL3) condition.

#### Generation of Chil3-Flp mice

The T7-transcribed Chil3_gRNA1 product, which was amplified by using KOD FX NEO (Toyobo) and the primers (Chil3_gRNA1_F 5’- TTAATACGACTCA CTATAGGttgcaagggcccttattgagGTTTTAGAGCTAGAAATAGCAAGTTAAAAT -3’; gRNA_ common_R2 5’- AAAAGCACCGACTCGGTGCCACTTTTTCAAGTTGATAACGGACTAGCCTTATTTTAACTTGCTATTTCTAGCTCT -3’) were used as the subsequent generation of Chil3_gRNA1. MEGAshortscript T7 (Thermo Scientific) was used for the generation of the gRNA. *Cas9* mRNA was generated by *in vitro* transcription (IVT) using mMESSAGE mMACHINE T7 ULTRA kit (Thermo Scientific) and the template that was amplified by PCR using pEF6-hCas9-Puro and the primers T7Cas9_IVT_F and Cas9_R and gel-purified. The synthesized gRNA and *Cas9* mRNA were purified using MEGAclear kit (Thermo Scientific). To generate Chil3-Flp mice, 1.4 kb fragment containing exon11 and 3’ untranslated sequence of the *Chil3* gene was amplified by PCR using primers Chil3_LA_F 5’- gaattcGTTTCAACAGGCTCAGTGGCTCAAGAAACATTTAGGAGGTGCCGTGGTCTG -3’; Chil3_LA_R 5’- ctcgagATAAGGCCTGCAACCTGAAACACAGAGAAAGACACAGCAATGACACTGGAG -3’; Chil3_RA_F 5’- ggatccGAGGAGCTTTACACAATGATTTGTCCTGAAATCTCAAATAAGATCAAGTTC -3’; Chil3_RA_R 5’- gcggccgcCCAAGGAATGTGTTGGATGGGAGGACCCTATTTCAACTACAATACCCAGGC -3’) and cloned in pBluescript vector. P2A peptide sequence containing mammalian codon optimized *Flp* recombinase cDNA, which were artificially synthesized and obtained from FASMAC, was inserted just before the stop codon of *Chil3* in the exon11. The targeting vector was gel purified and used for injection into embryos with Chil3_gRNA1 and *Cas9* mRNA. To obtain Chil3-Flp mice, CD45.2 C57BL/6 female mice (6-week-old) were superovulated and mated to CD45.2 C57BL/6 stud males. Fertilized one-cell-stage embryos were collected from oviducts and injected into the pronuclei or the cytoplasm with 100 ng/μl *Cas9* mRNA, 50 ng/μl gRNA and 50 ng/ml the targeting vector for Chil3-Flp mice. The injected live embryos were transferred into oviducts of pseudopregnant ICR females at 0.5 d post coitus. The male pup harboring the mutation was mated to CD45.2 C57BL/6 female mice and tested for the germ line transmission.

#### Microorganisms

*Mycobacterium abscesses* ATCC 19977 strain was cultured as previously described.^64^ *M. abs* was grown in 4.7 g/l Middlebrook 7H9 broth (BD) with 0.2% (v/v) glycerol and 10% (v/v) oleic acid-albumin-dextrose-catalase supplement (OADC, BD) at 37°C for 3 days. They were stored in 10% glycerol at -80°C. The quantification of mycobacterium was performed by plating 10-fold serial dilutions on 7H9 agar plates. *M. abs* expressing mCherry was generated based on pKRB1 vector.^68^ Briefly, the mCherry sequences were amplified by PCR with SalI and Hpal sites at 5’ and 3’ end. The fragment was switched with GFP sequence of the pKRB1 vector. The NEPA21 (1800 V) was used Transfection of plasmid into *M. abs*.

*S. pneumoniae* TIGR4 strain was cultured as previously described.^65^ *Spn* was grown overnight at 37°C with 5% CO_2_ in 37 g/l Todd-Hewitt Broth (Millipore) supplemented with 20 g/l Extract Yeast (Nacalai Tesque). They were stored in 30% glycerol at -80°C. The bacterium was cultured from a glycerol stock on one day before the infection experiment. It was then cultured for 2-3 h until it reached the exponential growth phase (OD_600_ = 0.4-0.5) and resuspended in PBS. Bacterial colony forming units (CFUs) was determined by plating 10-fold serial dilutions on TSA II 5% sheep blood agar plates (BD).

SARS-CoV-2 rB6MA, a C57BL/6 mouse-adapted SARS-CoV-2, was generated by a circular polymerase extension reaction,^69^ using SARS-CoV-2 NIID strain (2019-nCoV_Japan_TY_WK-5212020) as the backbone. rB6MA contains five mutations (Nsp4(H313Y), Nsp8(Q19R), Nsp13(P504S), M(H125Y), S(Q493K)) that were introduced into SARS-CoV-2 during serial passages of SARS-CoV-2 VIC2089 strain in C57BL/6 mice,^70^ as well as five mutations (Nsp12(P314L), N(R203K/G204R), S(N501Y, D614G)) that were originally introduced into the VIC2089 strain. Virus titer was estimated in fifty-percent tissue culture infective dose (TCID_50_) assay using Vero E6 cells for SARS-CoV-2.

### Method details

#### Infection and treatment

Mice were intraperitoneally injected with 100 mg/kg ketamine (Daiichi Sankyo Propharma) and 10 mg/kg xylazine (Elanco Japan), and then intranasally infected with *M. abs* (5x10^7^ CFU/mouse), *Spn* (3-4x10^7^ CFU/mouse) and SARS-CoV-2 (3x10^4^ TCID_50_/mouse).

For AM depletion regimen, 100 ng DT (Millipore) or PBS was administered intraperitoneally to CCD mice every 2-3 days for 3 days prior to infection.

For antibodies treatment, 1 mg/mouse rat IgG1 anti-IFN-γ antibody and rat IgG1 anti-HRP (Bio X cell) were administered intravenously on one day before infection.

For the prophylactic treatment regimen, 10 μg/mouse rmGM-CSF (PeproTech) in PBS/0.1% BSA or PBS/0.1% BSA alone was administered intranasally 6 hours prior to infection.^51^

#### Determination of bacterial loads in lung with *Mycobacterium* and pneumococcus

For *Mycobacterium*, the infected lungs were homogenized in culture tubes containing 1 ml of 7H9 medium on ice at 12500 rpm by PT1300D homogenizer (Kinematica AG). To count bacterial CFU, tissue suspensions diluted 10-fold as appropriate were seeded in 7H9 agar plates of 100 μl each and incubated at 37°C.

For pneumococcus, BALF was collected from infected mice by injecting 1 ml of PBS. To count bacterial CFU, the BALF diluted 10-fold as appropriate were seeded in TSA II 5% sheep blood aga plates of 20 μl each and incubated at 37°C with 5% CO_2_.

#### Determination of viral loads in lung with SARS-CoV-2

CCD mice were treated with PBS or DT at indicated timing, then infected with rB6MA. Mice were euthanized at three days post infection and lung were isolated. Lysates from each lung were serially diluted by 10-fold and cultured in Vero-E6 cells and cultured for 3 days. Virus titer in each were estimated by TCID_50_.

#### Various organ cell preparation

All organs were collected from mice after sufficient PBS perfusion and were processed according to the protocol^71^ as follows.

##### Lung

Razor-chopped lungs were digested with 1 mg/ml Collagenase D (Roche), 80 μg/ml Dispase II (Roche), 20 μg/ml DNase I (Roche) for 60 min at 37°C with shaking. The sample was washed with PBS after passing through a 70 μm cell strainer. It was suspended in 1 ml ACK buffer and kept at RT for 2-3 min to lyse red blood cells, then washed in PBS and the cells were counted on a blood cell counter.

##### Liver and kidney

The organs were digested in the same way as the lungs. The digested tissues were suspended in 40% Percoll (Sigma) and centrifuged at 2380xg for 10 min to remove debris. The cell pellets were treated with ACK buffer and then washed with PBS.

##### Skin

Ears were cut and digested with 2 mg/ml Dispase II for 90 min at 37°C. Skins cut with scissors were digested with 1 mg/ml Collagenase D and 20 μg/ml DNase I for 30 min at 37°C. The digested tissue was passed through an 18G needle hole. The sample was washed with PBS after passing through a 70 μm cell strainer.

##### Brain

Razor-chopped brains were mashed in PBS on a 70 μm cell strainer using syringe plunger. The filtrated tissue was suspended in 40% Percoll and centrifuged at 2380xg for 20 min to remove debris. The cell pellets were treated with ACK buffer and then washed with PBS.

##### Small intestine and colon

Intestines were opened, feces removed and shaken in HBSS with 5 mM EDTA for 20 min at 37°C. After removal of mucus and muscle layers, samples were digested with 1 mg/ml Collagenase D, 500 μg/ml Dispase II (gibco) and 200 μg/ml DNase I for 10-15 min. The digested tissues were suspended in 40% Percoll, and 80% Percoll was carefully added to the lower layer using a Pasteur pipette. They were centrifuged at 700xg for 20 min, and the cells at the border between 40% and 80% Percoll were collected. The cells were treated with ACK buffer and then washed with PBS.

##### Bone marrow, spleen and lymph nodes

After cutting both ends of the femur and tibia, bone marrow cells were collected by injecting RPMI 1640 with a syringe with a 26G needle. Bone marrow cell, Spleen and lymph nodes were mashed in PBS on a 70 μm cell strainer using syringe plunger. The filtrated tissues were treated with ACK buffer and then washed with PBS.

#### PBMCs and peritoneal exudate cells (PEC)

Blood samples were collected from the orbital venous plexus and were prevented from coagulation in PBS containing 5 mM EDTA. PECs were collected by injecting RPMI 1640 into the abdominal cavity before the mice were opened. The whole blood was treated with 10 ml of ACK buffer for 10 min and PEC with 1 ml of ACK buffer for 2 min, then washed with PBS.

##### BMDMs

BMDMs were differentiated in RPMI 1640 (Nacalai Tesque) containing 10% heat-inactivated fetal bovine serum (gibco), 50 mM 2-Mercaptoethanol (Nacalai Tesque), 100 U/ml penicillin and 0.1 mg/ml streptomycin (Nacalai Tesque) and 30% L929 cell (ATCC) supernatant for 6 days, then maintained in RPMI 1640 with 5% L929 cell supernatant under the same conditions. On day 6-7, the cells were cultured for an additional 3 days at 37°C in the presence or absence of recombinant murine IL-4 (50 ng/ml, peprotech). After 3 days of culture, flow cytometry and RNA extraction were performed.

#### Flow cytometry and cell soring

The cell samples (10^5^-10^6^ cell) were resuspended 1/200 purified anti-mouse CD16/32 (Biolegend) diluted 1/200 in FACS buffer (PBS, 3% FBS, 1 mM EDTA and 0.1% NaN_3_ sodium azide) for 5 min on ice. Antibodies cocktails were diluted into 1/100, and 20 μl was added to cells for 15 min on ice. Cells were washed twice by FACS buffer. Flow cytometry and cell sorting were performed using an Attune NxT flow cytometer (Thermo Scientific) and FACS Aria III (BD), respectively.

#### RNA sequencing

Total RNA was extracted from the sorted cells using RNeasy Mini Kit (Qiagen) according to the instructions. Full length cDNA was generated using a SMART-Seq HT Kit (Takara Bio) according to the manufacturer's instructions. An Illumina library was prepared using a Nextera DNA Library Preparation Kit (Illumina) according to the SMARTer kit instructions. Sequencing was performed on an Illumina NovaSeq 6000 sequencer (Illumina) in 101-base single-end mode. Sequenced reads were mapped to the mouse reference genome sequences (mm10) using TopHat v2.1.1 in combination with Bowtie2 ver. 2.2.8 and SAMtools ver. 0.1.18. The fragments per kilobase of exon per million mapped fragments (FPKMs) was calculated using Cufflinks version 2.2.1. Raw count data were analyzed using iDEP.96 with normalized FPKM in alveolar macrophage > 100 and |FPKM fold change| > 2.0 in were considered as differentially expressed genes (DEGs). Candidate genes were narrowed down using BioGPS to those with low gene expression in organs other than the lung (particularly in the brain and intestinal tract with expression levels below 30). Then, candidate genes were further narrowed down by comparison with previous reports of RNA sequencing in alveolar macrophages.

#### Quantitative PCR

After RNA extraction as described above, RNA was reverse transcribed using Verso cDNA synthesis Kit (Thermo Scientific) according to the instructions. Quantitative PCR was performed on a CFX Connect (Bio-Rad) using GoTaq qPCR Master Mix (Promega) and primers described below. Relative mRNA expression levels were normalized by β-actin and 2^-ΔΔCT^ values were calculated.

#### Immunohistochemical staining

10% formalin was administered transtracheally to fix the lungs, and post-fixed with 10% formalin at 4°C for 5 h. The fixed lung was dehydrated with 30% sucrose/PBS solution (w/w) at 4°C overnight. The sample was embedded in FSC22 Frozen Section Media (Leica) and sectioned at 10 μm using a CM1860 UV (Leica). The section was treated with 0.25% Triton X-100 for 10 min at RT and washed three times with 0.1% PBST. The sample was blocked in blocking buffer (0.1% PBST with 0.1% BSA and 1% mouse serum) for 1 h at RT and then incubated with goat anti DTR polyclonal antibody (R&D systems) diluted into 1/100 in blocking buffer at 4°C overnight. The section was washed three times and incubated with 1/1000 CF488A Donkey anti-goat IgG H&L (Biotium) and 1/250 BV421 rat anti-mouse SiglecF (BD) in blocking buffer for 1 h at RT. The stained sample was washed three times and were sealed in Aqueous Mounting Medium, PermaFluor (Fisher Scientific). The images were observed using FV3000 (Olympus).

#### Total protein determination in bronchoalveolar lavage fluid (BALF)

The turbidity of BALF was measured at OD_600_ with a NanoDrop-2000C (Thermo Scientific). Two-fold dilution series of BALF and CBB solution (Nacalai Tesque) were reacted according to the instructions. After the incubation, the plate was read at 595 nm in an iMark Microplate Absorbance Reader (Bio-Rad), and total protein amount was estimated by calibration curves with a dilution series of BSA (2000-15.6 μg/ml).

### Quantification and statistical analysis

We compared two groups by unpaired two-tailed Student’s t test and multiple experimental groups by one-way ANOVA with Turkey multiple comparisons or two-way ANOVA with sidak's multiple comparisons test using GraphPad Prism 10.0. Statical significance value are shown as “N.S.” for not significant, ∗*p* < 0.05, ∗∗*p* < 0.01 and ∗∗∗*p* < 0.001.
