# Supplementary material for: Targeted labeling and depletion of alveolar macrophages using VeDTR mouse technology
Source: iScience. 2025 Feb 9;28(3):111975. doi: 10.1016/j.isci.2025.111975 (PMC11889737; doi:10.1016/j.isci.2025.111975)
Supplement: Document S1. Figures S1–S6 [file mmc1.pdf]

iScience, Volume *28*

Supplemental information

Targeted labeling and depletion of alveolar macrophages using VeDTR mouse technology

Yuki Nakayama, Miwa Sasai, Ayumi Kuratani, Masaaki Okamoto, Daisuke Okuzaki, Kentaro Yamamoto, Chikako Ono, Masaya Yamaguchi, Shigetada Kawabata, Noriko Shinjyo, Yasutaka Okabe, Yoshiharu Matsuura, Manabu Ato, and Masahiro Yamamoto

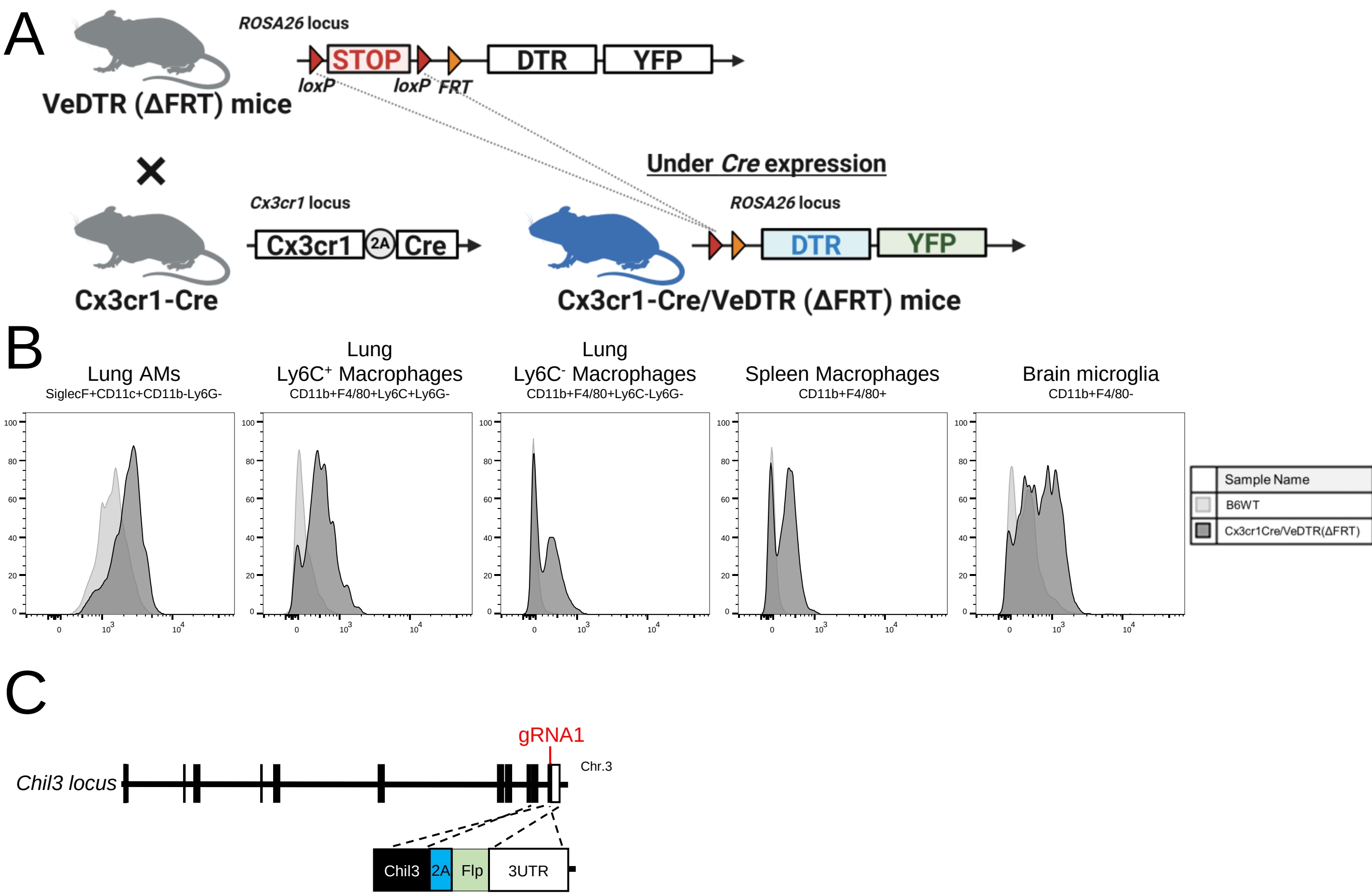

Fig. S1. Supplemental data on the generation of CCD mice. Related to Fig. 1

(A) Schematic of the principle of gene expression in Cx3cr1Cre/VeDTR (ΔFRT) mice. DTR and YFP are expressed in cells expressing Cx3cr1-Cre in these mice, as in Cx3cr1-Cre-DTR mice.

(B) The identification of YFP^{+} cells in lung, spleen, and brain macrophages of WT (gray, n = 3) and Cx3cr1-Cre/VeDTR (ΔFRT) mice (black, n = 3).

(C) Schematic diagram of genetic manipulation for the creation of Chil3-Flp mice.

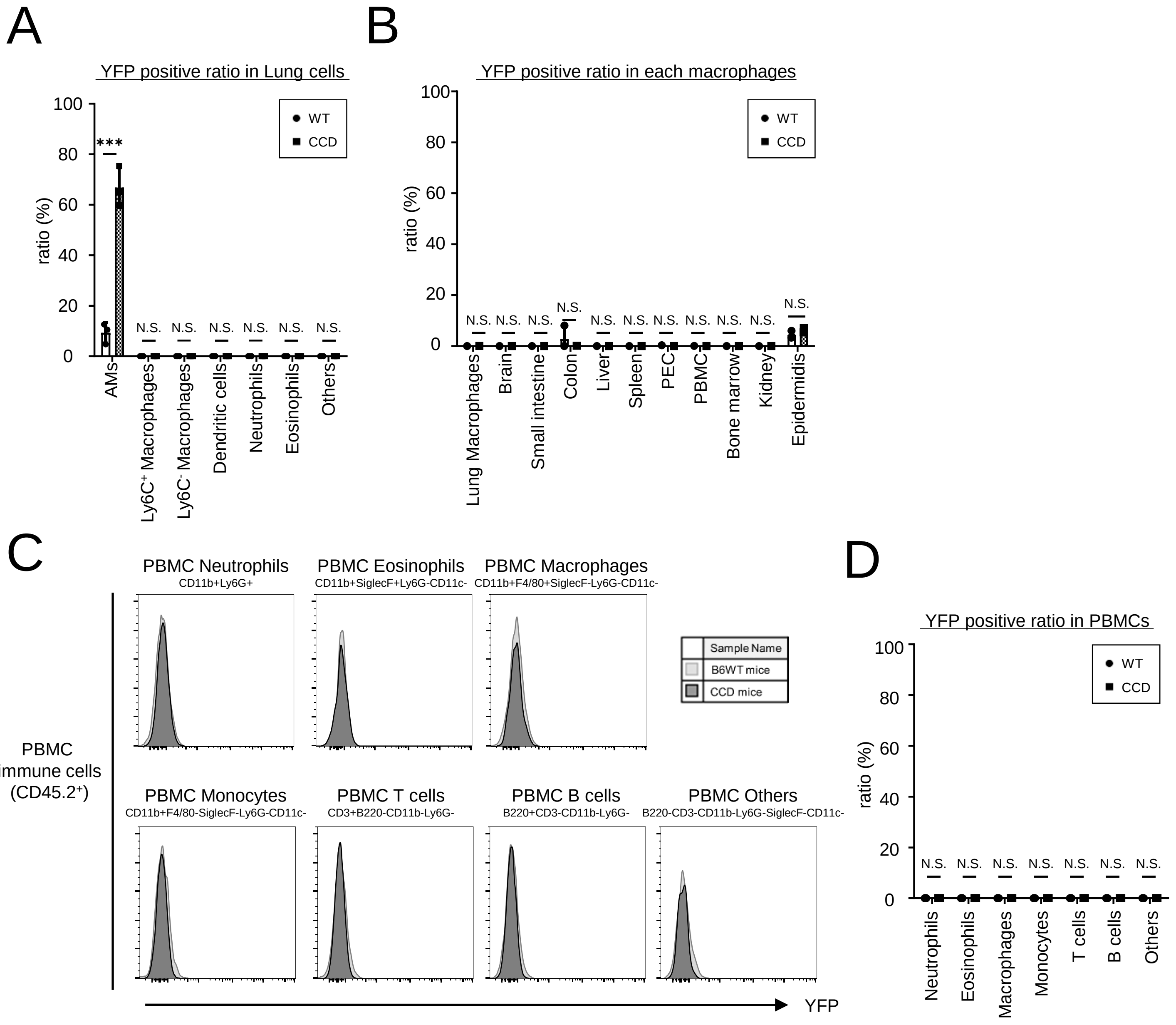

Fig. S2. The identification of YFP⁺ cells in macrophage and blood cells of uninfected CCD mice. Related to Fig. 1

(A and B) Statistical analysis of the YFP-positive rate in lung cells (A) and macrophages of systemic organs (B) in WT (circle n = 3) and CCD mice (square, n = 3). Fig. S2A and S2B correspond to the results of Fig. 1F and 1G, respectively.

(C) Flow cytometric analysis showing YFP positivity in PBMCs in WT (gray, n = 3) and CCD mice (black, n = 3).

(D) Statistical analysis of YFP-positive rates in Fig. S2C.

Statistical significance assessment: two-way ANOVA with sidak's multiple comparisons test (A, B and D). Error bars represent SD. *p<0.05, **p<0.01, ***p<0.001, N.S.: not significant.

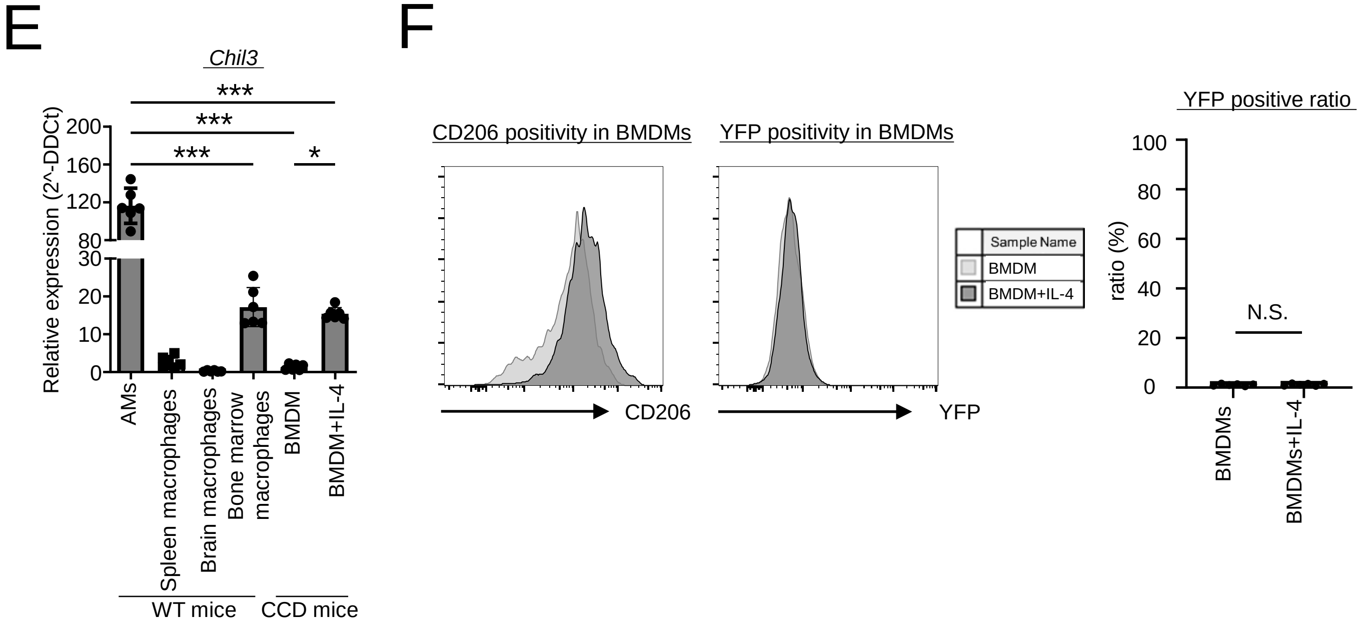

Fig. S2 (continued).

(E) RT-qPCR analysis of *Chil3* gene expression with *β-actin* as the endogenous control in macrophages from various organs of WT mice and BMDMs from CCD mice (n = 3 each). AMs (SiglecF^{+}CD11c^{+}), splenic macrophages (CD11b^{+} Ly6G^{-}), brain macrophages (CD11b^{+} Ly6G^{-}), and bone marrow macrophages (CD11b^{+} Ly6G^{-}) were sorted and collected from WT mice. BMDMs from CCD mice were stimulated with IL-4 for 3 days to induce M2 macrophages.

Expression level was calculated using the comparative cycle threshold ($2^{-\Delta\Delta Ct}$) value method, with the expression level of BMDMs from CCD mice set to 1.0 as the reference.

(F) Flow cytometric analysis of BMDMs from CCD mice induced to M2 macrophages by IL-4 stimulation (n = 3 each). The left plot shows the percentage of CD206 and YFP positive cells in BMDMs stimulated with IL-4 (black) or unstimulated (gray). The right graph shows statistical analysis of the YFP-positive cell percentage. The BMDMs in F correspond to the cells in E. Data are representative of two independent experiments.

Statistical significance assessment: two-way ANOVA with sidak's multiple comparisons test (A and B), one-way ANOVA with Tukey's multiple comparisons test (E) and unpaired two-tailed Student’s t test (F). Error bars represent SD. *p<0.05, ***p<0.001, N.S.: not significant.

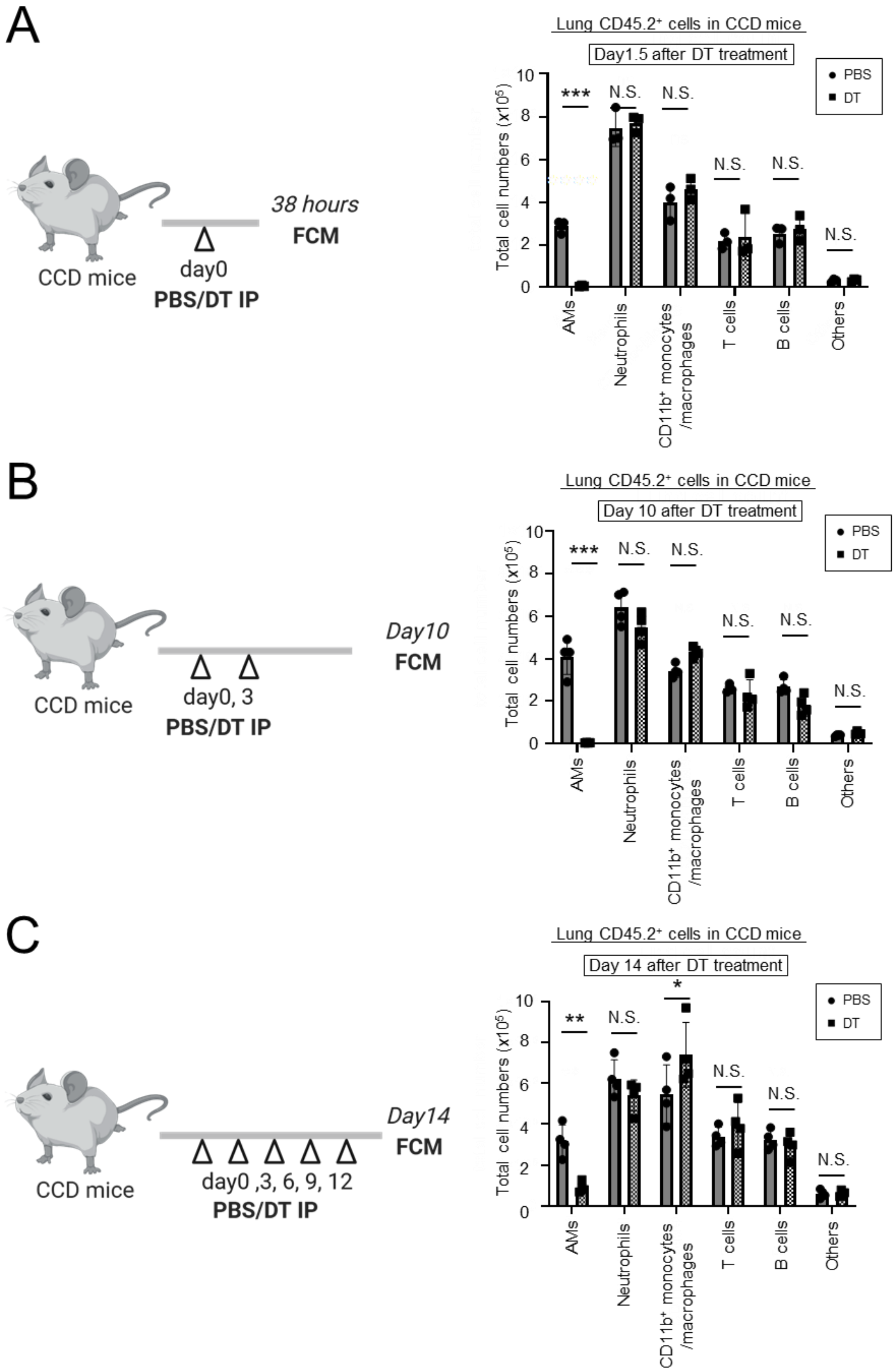

Fig. S3. Supplemental data on Selective depletion of AMs with DT in CCD mice. Related to Fig. 2

(A) Number of cells in CCD mouse lungs 38 hours after single PBS (circle) or DT (square) administration (n=3 each).

(B) Number of cells in CCD mouse lungs 10 days after two PBS (circle) or DT (square) administration (n = 4 each).

(C) Number of cells in CCD mouse lungs 14 days after five PBS (circle) or DT (square) administrations (n=4 each).

The diagram on the left shows the experimental schedule. The black arrows in the diagram indicate the time of PBS or DT treatment (A-C in common).

Statistical significance assessment: two-way ANOVA with sidak's multiple comparisons test (A, B and C). Error bars represent SD. *p<0.05, **p<0.01, ***p<0.001, N.S.: not significant.

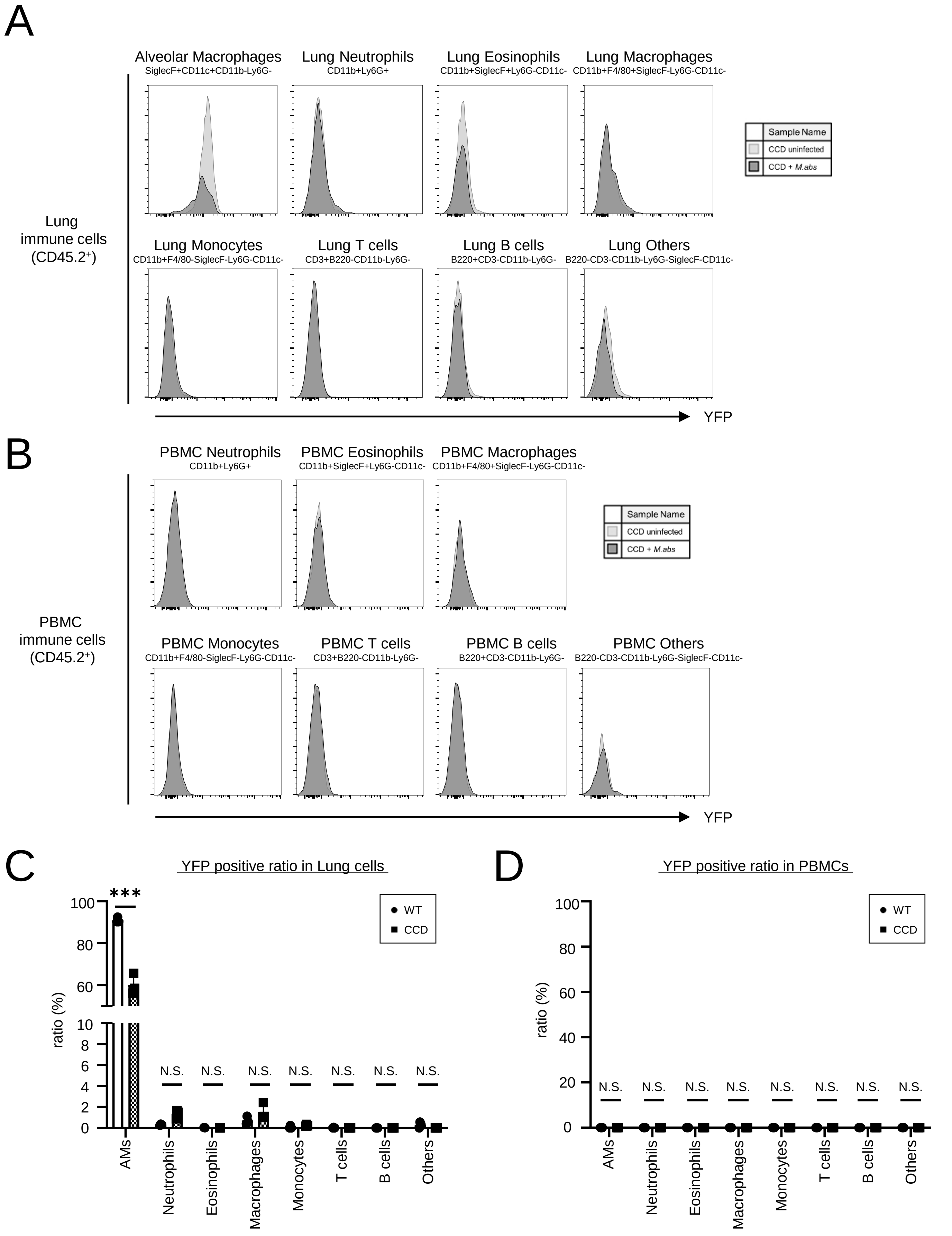

Fig. S4. The identification of YFP⁺ cells in lung and blood cells of CCD mice infected with *M.abs.* Related to Fig. 3

(A and B) Flow cytometric analysis showing YFP positivity in lung cells (A) and macrophages of PBMCs (B) in uninfected CCD mice (gray, n = 3) and CCD mice infected with *M.abs* for 7 days (black, n = 3).

(C and D) Statistical analysis of YFP-positive rates in Fig. S4A and S4B, respectively.

A

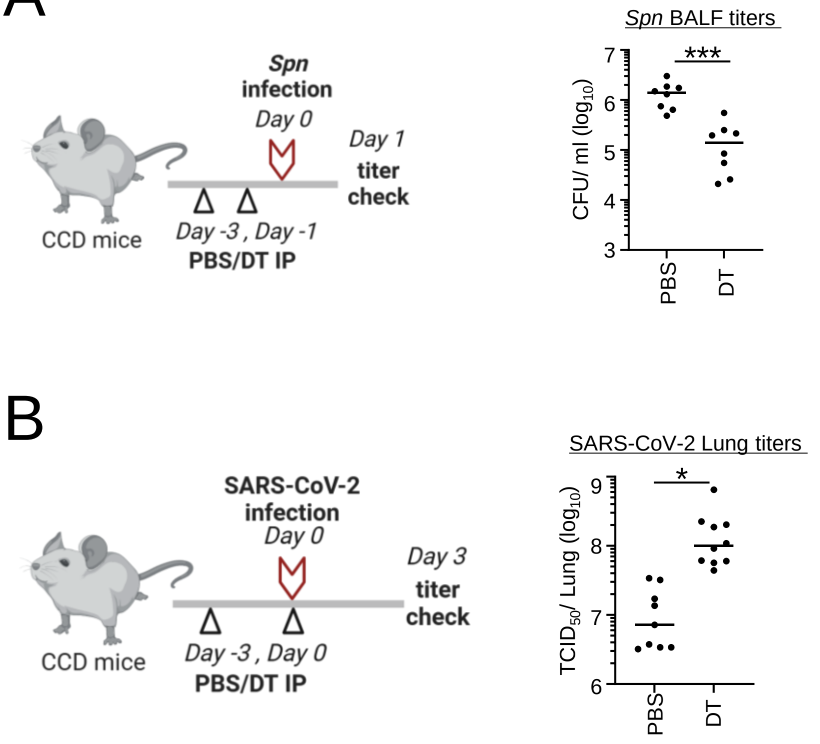

Fig. S5. The role of AMs in various respiratory infections. Related to Fig. 3

(A) *Streptococcus pneumoniae* bacterial load on one day of intranasal infection in BALF of CCD mice pretreated with PBS or DT (n = 8 each). The diagram on the left shows the experimental schedule. The black and red arrows in the diagram indicate the time of PBS or DT treatment and infection, respectively (A and B in common). (B) SARS-CoV-2 (B6 adapted MA10) viral load on days 3 of intranasal infection in the lungs of CCD mice pretreated with PBS or DT (n = 9 each).

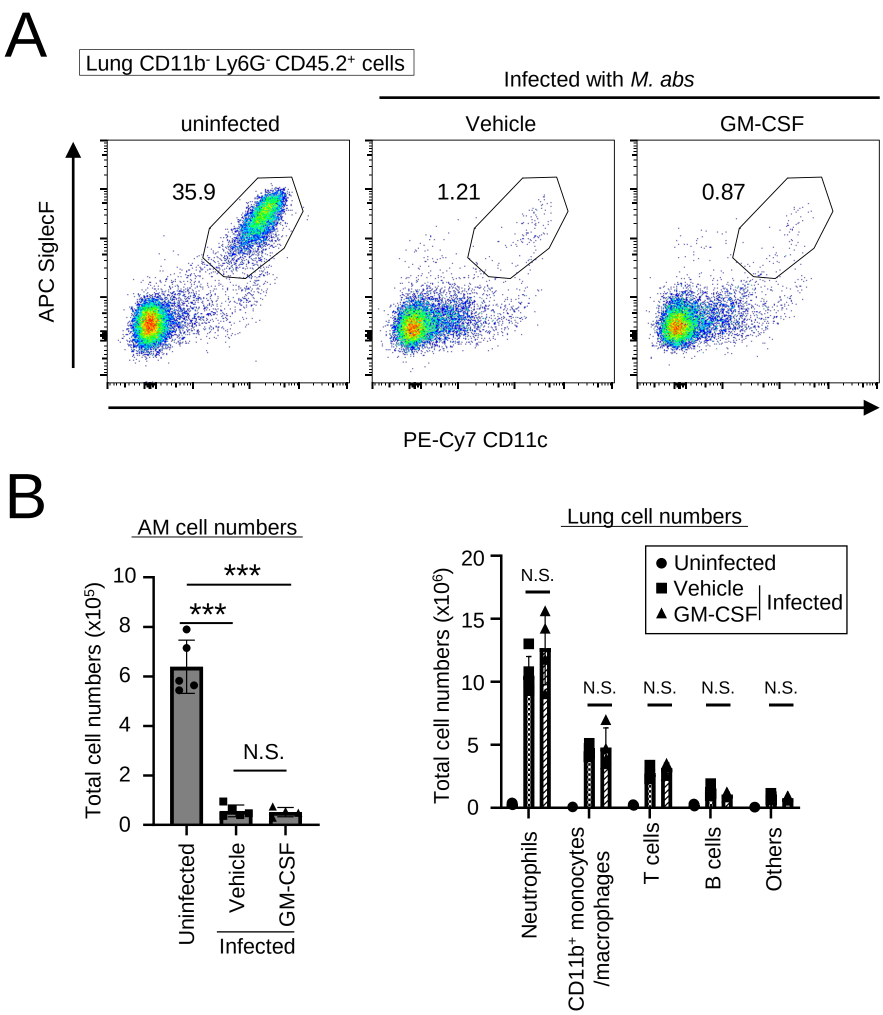

Fig. S6. GM-CSF treatment does not prevent AM disappearance during *M. abs* infection. Related to Fig. 5

(A and B) Flow cytometric analysis of AMs in the lungs of WT mice 7 days after *M. abs* infection. Mice were pre-treated intranasally with either vehicle (0.1% BSA) or recombinant mouse GM-CSF (n = 4–5 each) 6 h prior to infection. Results are presented as a plot diagram (A) and lung cell count (B). The numbers in the plot diagram indicate the percentage of AMs among CD11b^{-} Ly6G^{-} CD45.2^{+} cells.
